# Gaps in childhood immunizations and preventive care visits during the COVID-19 pandemic: a population-based cohort study of children in Ontario and Manitoba, Canada, 2016–2021

**DOI:** 10.17269/s41997-023-00797-y

**Published:** 2023-07-13

**Authors:** Andrea Evans, Alyson L. Mahar, Bhumika Deb, Alexa Boblitz, Marni Brownell, Astrid Guttmann, Therese A. Stukel, Eyal Cohen, Joykrishna Sarkar, Nkiruka Eze, Alan Katz, Tharani Raveendran, Natasha Saunders

**Affiliations:** 1 Children’s Hospital of Eastern Ontario, Ottawa, ON Canada; 2 Department of Pediatrics, University of Ottawa, Ottawa, ON Canada; 3 ICES, Toronto, ON Canada; 4 Department of Community Health Sciences, University of Manitoba, Winnipeg, MB Canada; 5 Manitoba Centre for Health Policy, Winnipeg, MB Canada; 6 Children’s Hospital Research Institute of Manitoba, Winnipeg, MB Canada; 7 The Hospital for Sick Children, Toronto, ON Canada; 8 Child Health Evaluative Sciences, SickKids Research Institute, Toronto, ON Canada; 9 Department of Pediatrics, Temerty Faculty of Medicine, University of Toronto, Toronto, ON Canada; 10 Institute of Health Policy, Management and Evaluation, University of Toronto, Toronto, ON Canada; 11 Edwin S.H. Leong Centre for Healthy Children, University of Toronto, Toronto, ON Canada; 12 Department of Family Medicine, University of Manitoba, Winnipeg, MB Canada

**Keywords:** COVID-19, Immunization coverage, Health systems, Pediatrics, Primary care, Preventive care, Family medicine, Cohort study, Well-child care, COVID-19, couverture vaccinale, systèmes de santé, pédiatrie, soins primaires, soins préventifs, médecine familiale, études de cohortes, services de consultation pédiatrique

## Abstract

**Objective:**

We aimed to estimate the changes to the delivery of routine immunizations and well-child visits through the pandemic.

**Methods:**

Using linked administrative health data in Ontario and Manitoba, Canada (1 September 2016 to 30 September 2021), infants <12 months old (N=291,917 Ontario, N=33,994 Manitoba) and children between 12 and 24 months old (N=293,523 Ontario, N=33,001 Manitoba) exposed and unexposed to the COVID-19 pandemic were compared on rates of receipt of recommended a) vaccinations and b) well-child visits after adjusting for sociodemographic measures. In Ontario, vaccinations were captured using physician billings database, and in Manitoba they were captured in a centralized vaccination registry.

**Results:**

Exposed Ontario infants were slightly more likely to receive all vaccinations according to billing data (62.5% exposed vs. 61.6% unexposed; adjusted Relative Rate (aRR) 1.01 [95% confidence interval (CI) 1.00-1.02]) whereas exposed Manitoba infants were less likely to receive all vaccines (73.5% exposed vs. 79.2% unexposed; aRR 0.93 [95% CI 0.92-0.94]). Among children exposed to the pandemic, total vaccination receipt was modestly decreased compared to unexposed (Ontario aRR 0.98 [95% CI 0.97-0.99]; Manitoba aRR 0.93 [95% CI 0.91-0.94]). Pandemic-exposed infants were less likely to complete all recommended well-child visits in Ontario (33.0% exposed, 48.8% unexposed; aRR 0.67 [95% CI 0.68-0.69]) and Manitoba (55.0% exposed, 70.7% unexposed; aRR 0.78 [95% CI 0.77-0.79]). A similar relationship was observed for rates of completed well-child visits among children in Ontario (aRR 0.78 [95% CI 0.77-0.79]) and Manitoba (aRR 0.79 [95% CI 0.77-0.80]).

**Conclusion:**

Through the first 18 months of the pandemic, routine vaccines were delivered to children < 2 years old at close to pre-pandemic rates. There was a high proportion of incomplete well-child visits, indicating that developmental surveillance catch-up is crucial.

**Supplementary Information:**

The online version contains supplementary material available at 10.17269/s41997-023-00797-y.

## Résumé

**Objectif:**

Nous avons voulu estimer les changements dans l’administration des vaccins de routine et dans les consultations pédiatriques pendant la pandémie.

**Méthode:**

À l’aide des données administratives sur la santé couplées de l’Ontario et du Manitoba, au Canada (1^er^ septembre 2016 au 30 septembre 2021), nous avons comparé les taux de réception : a) des vaccins recommandés et b) des consultations pédiatriques recommandées pour les nourrissons de < 12 mois (N = 291 917 en Ontario, N = 33 994 au Manitoba) et pour les enfants de 12 à 24 mois (N = 293 523 en Ontario, N = 33 001 au Manitoba) exposés et non exposés à la pandémie de COVID-19, après ajustement en fonction de mesures sociodémographiques. En Ontario, les vaccins ont été saisis à l’aide de la base de données des factures des médecins; au Manitoba, ils ont été saisis dans un registre de vaccination centralisé.

**Résultats:**

Les nourrissons exposés en Ontario étaient légèrement plus susceptibles de recevoir tous les vaccins selon les données de facturation (62,5 % pour les nourrissons exposés c. 61,6 % pour les nourrissons non exposés; risque relatif ajusté [RRa] 1,01 [intervalle de confiance (IC) de 95 % 1,00-1,02]), tandis que les nourrissons exposés au Manitoba étaient moins susceptibles de recevoir tous les vaccins (73,5 % pour les nourrissons exposés c. 79,2 % pour les nourrissons non exposés; RRa 0,93 [IC de 95 % 0,92-0,94]). Chez les enfants exposés à la pandémie, le total des vaccins reçus était un peu plus faible que chez les enfants non exposés (RRa en Ontario 0,98 [IC de 95 % 0,97-0,99]; RRa au Manitoba 0,93 [IC de 95 % 0,91-0,94]). Les nourrissons exposés à la pandémie étaient moins susceptibles d’avoir eu toutes les consultations pédiatriques recommandées en Ontario (33 % pour les nourrissons exposés, 48,8 % pour les nourrissons non exposés; RRa 0,67 [IC de 95 % 0,68-0,69]) comme au Manitoba (55 % pour les nourrissons exposés, 70,7 % pour les nourrissons non exposés; RRa 0,78 [IC de 95 % 0,77-0,79]). Une relation semblable a été observée pour les taux de consultations pédiatriques complètes chez les enfants en Ontario (RRa 0,78 [IC de 95 % 0,77-0,79]) et au Manitoba (RRa 0,79 [(IC de 95 % 0,77-0,80]).

**Conclusion:**

Au cours des 18 premiers mois de la pandémie, les vaccins de routine ont été administrés aux enfants de < 2 ans à des taux proches de ceux d’avant la pandémie. Il y a eu une forte proportion de consultations pédiatriques incomplètes, ce qui indique qu’il est essentiel de rattraper la surveillance du développement.

## Introduction

The coronavirus disease 2019 (COVID-19) pandemic contributed to disruption of the delivery of core preventive child health services including routine immunizations and well-child visits (Causey et al., 2021). Disruptions of immunizations services, even for discrete time periods, can result in increased likelihood of vaccine-preventable disease outbreaks (WHO, 2020). An under-vaccinated population related to vaccination disruption from COVID-19 has been a cause of concern for re-emerging vaccine-preventable diseases such as polio (Rigby, 2022). In addition, routine well-child visits provide health supervision, anticipatory guidance, screening, and management of acute and chronic conditions, and care coordination. Early identification and intervention is important in many childhood conditions such as in developmental delays whereby missed or delayed diagnosis can have long-term health consequences (Canadian Task Force on Preventive Health Care, 2016). When routine appointments are not accessible or delivered virtually, parents may defer care or seek health services in emergency departments with consequences to the healthcare system and child health outcomes.

Globally, marked disruptions to routine immunizations were documented in the first year of the pandemic, with measles and tetanus vaccination coverage reduced by over 7% (Causey et al., 2021). In Canada, studies from different provinces have shown a similar reduction in vaccine uptake within the first year of the pandemic (Dong et al., 2022; Ji et al., 2022; Kiely et al., 2021; Lee et al., 2022; MacDonald et al., 2022; Sell et al., 2021). Reports of delayed and missed vaccinations caused public health concerns resulting in Canada’s National Advisory Committee on Immunization recommending prioritizing primary immunization series up to 18 months old during the pandemic, and provinces and territories implementing vaccine catch-up programs (NACI, 2020; Allan et al., 2021).

In both Ontario and Manitoba, we have previously observed a large shift to virtual primary care during the pandemic and an initial decline with some recovery in the rates of well-child visits (Glazier et al., 2021; Saunders et al., 2021). The effects of such large shifts from office to virtual care on routine immunizations and recommended well-child visits are unknown, particularly after allowing for a period of catch-up (Saunders et al., 2021).

We aim to quantify differences in immunization and preventive care visits completed before 12 months of age, and between 12 and 24 months of age, for children post COVID-19 pandemic onset, compared to children before pandemic onset in two Canadian provinces, Manitoba (population ~1.4 million) and Ontario (population ~15 million). We capture immunization and well-child visit rates up until September 2021. Due to the disrupted delivery of services, we hypothesized that the proportion of infants and children who received all vaccines or well-child visits would be lower if they were exposed to the pandemic compared to unexposed infants and children. Our secondary objective was to describe and quantify changes in the receipt of individual vaccines and well-child visits.

## Methods

### Study design, setting, and population

**Fig. 1:**
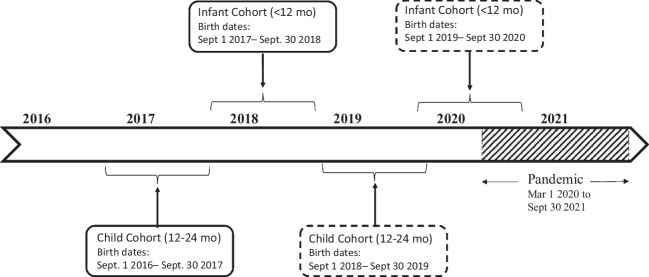
Definition of infant (< 12 months old) and children (12-24 months old) cohorts. Exposed cohort (subjects with recommended immunizations and visits post-pandemic onset) are in dashed lines. Unexposed cohort (subjects with all recommended immunizations and visits pre-pandemic onset) are in continuous lines. Exposure to the pandemic defined as 6 months or more (COVID-19 pandemic defined as 1 March 2020 to 30 September 2021)

This was a population-based cohort study of children 0 to 24 months old, born between 2016 and 2020 in Ontario and Manitoba, using linked health and administrative data. The exposed cohort had all vaccines and visits after the pandemic onset and was exposed to at least 6 months of the pandemic, as defined from 1 March 2020 to 30 September 2021, and therefore born between 1 September 2018 and 30 September 2020. The unexposed cohort had all visits and vaccinations prior to the pandemic onset and completed prior to 17 March 2020, thus were born between 1 September 2016 and 30 September 2018. The last date of data collection was 30 September 2021. Pre-pandemic (unexposed) and post-pandemic (exposed) infant and child cohort definitions by birth date are illustrated in Fig. 1.

**Fig. 2:**
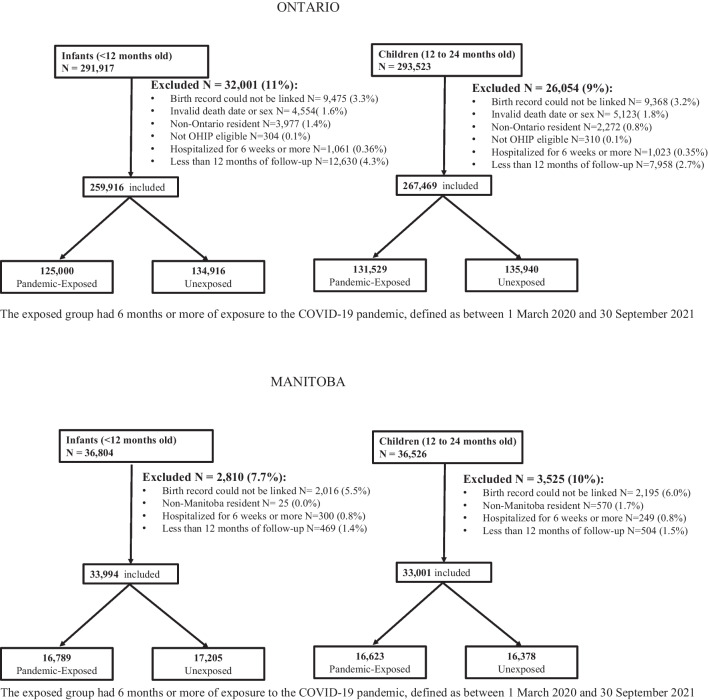
Flowsheet of participant inclusions and exclusions

Cohort numbers and exclusions are shown in Fig. 2. Infants were excluded if their demographic information (birthdate and sex) was unknown, if they were not Ontario/Manitoba residents at the time of birth and therefore not eligible for coverage through the Ontario/Manitoba Health Insurance Plan, if they were hospitalized at birth for greater than 6 weeks, if their birth records could not be reliably linked (invalid sex or linkage number), or if there was less than 12 months of follow-up.

### Data sources

We utilized health administrative and demographic databases housed and linked at ICES in Ontario and the Manitoba Centre for Health Policy. See Supplementary Table 1 for databases and their content. Individual-level records were linked by unique encoded identifiers derived from the health care numbers of individuals eligible for provincial health insurance coverage. We used demographic information (date of birth, sex and postal code) from provincial health insurance registries (Ontario’s Registered Persons Database, Manitoba Health Insurance Registry) and physician billings databases (Ontario Health Insurance Plan, Manitoba Medical Services) to ascertain outpatient physician visits to family physicians and pediatricians.

Childhood immunizations and well-baby visits are largely administered in outpatient settings through family physicians/general practitioners and pediatricians in Ontario. In Manitoba, vaccinations and well-baby visits are additionally administered by public health nurses, particularly in rural health regions (Hilderman et al., 2011). In Ontario, vaccination data are captured by billing claims. In September of 2011, vaccine-specific billing codes were introduced. A validation study showed high positive predictive value and high specificity, with moderate sensitivity (Schwartz et al., 2015). Unlike in Ontario, Manitoba’s immunization data are in a centralized immunization registry and thus not dependent on provider billing completeness and accuracy. In Manitoba, vaccination rates were defined using the Public Health Information Management System (PHIMS) data. See Supplementary Table 2 for codes used for Ontario and Manitoba.

### Outcomes

#### Vaccinations

As per the Ontario Immunization Schedule, infants should receive 3 doses of the diphtheria, tetanus, pertussis, polio, and haemophilus influenzae type B (DTaP-IPV-HiB) vaccines and 2 doses of the pneumococcal conjugate 13 (Pneu-C-13) vaccine in the first 6 months of life. The recommendations are that children receive one additional dose of Pneu-C-13, measles, mumps and rubella (MMR) and meningococcal type C (Men-C-C) at 12 months, along with a dose of varicella at 15 months and DTap-IPV-HiB at 18 months. The Manitoba vaccine schedule recommended by Manitoba Health has the same recommendations with the following exception: the MMR vaccine is combined with the varicella vaccine (MMRV). Manitoba immunization data include data on Rotavirus immunization, but these data were not available in Ontario and thus not reported.

For infants, our primary outcome was receipt of 3 doses of DTaP-IPV-HiB and 2 doses of Pneu-C-13 by the age of 12 months. For children, our primary outcome was receipt of 1 dose of Pneu-C-13, MMR or MMRV (in Manitoba), and Varicella (in Ontario), Men-C-C, and DTap-IPV-HiB between 12 and 24 months. In Ontario, only vaccinations that were billed by a physician were captured. No minimum time period was required between vaccine doses. Supplementary Figures 1 and 2 illustrate distribution of age of receipt for each vaccine. In both age groups, we allowed 6 months from the last recommended dose timing to allow for vaccine catch-up. Secondary outcomes were the proportion of recommended doses for individual vaccines.

#### Well-child visits

In Ontario, well-child visits occur at 2, 4, 6, 9, 12, 15, and 18 months of age as recommended by the Rourke Baby Record (Rourke & Rourke, 1985), along with recommendations for an ‘enhanced’ 18-month visit (Williams et al., 2008). Enhanced 18-month visits in Ontario occur in lieu of a well-baby visit, and contain a more comprehensive developmental assessment and have a unique billing code. In Manitoba the recommendations are similar, but without a routine recommendation for a 15-month visit nor the option for an ‘enhanced’ 18-month visit (Doctors Manitoba, 2021). From both primary and secondary outcomes, well-child visits that occurred within the first 30 days of life were excluded as these were considered newborn visits, which vary in frequency and indication. In Ontario, a small proportion (<1%) of primary care is provided through community health centres, salaried physicians and/or nurse practitioners who may shadow bill. Due to data limitations, these visits were not included. In Manitoba, although vaccinations by public health nurses were captured, well-baby visits by the same public health nurses were not captured in physician billing databases and thus not reported in this study.

For infants, our primary outcome was receipt of 4 well-child visits by 12 months of age in Ontario and Manitoba.

For children 12 to 24 months in Ontario, we defined the primary outcome as completion of 3 well-child visits (including the 18-month enhanced visit) and in Manitoba, this primary outcome was defined as 2 well-child visits between 12 and 24 months of age. We also report the average number of visits completed. Any well-baby visit, whether virtual or not, was captured.

### Covariates

Demographic data captured included maternal age and parity, area of residence (urban vs rural), socioeconomic measures such as neighbourhood income quintile, and provincial health region. The primary care practitioner who provided the majority of primary care to the child was determined using the physician billing codes listed in Supplementary Table 2. Primary care affiliation was classified as family physician/general practitioner, pediatrician, primary care nurse, and no assigned primary care provider. Continuity of care was provided for descriptive purposes but not used in the model, and was defined as those where ≥ 76% of all visits were to their assigned provider. Covariates included in the model were chosen that had a known association with receipt of health services such as vaccination (Chiem et al., 2022; Glazier et al., 2015; Saunders et al., 2021; Walton et al., 2022).

### Analysis

We conducted Ontario and Manitoba analyses separately because individual-level information cannot be combined across provinces. Demographic and baseline characteristics were reported using percentages, mean, median, standard deviations and inter-quartile range (IQR).

We generated Poisson models to compare infants who received their vaccinations and well-child visits during the pandemic compared to those pre-pandemic. Relative risks (aRR, 95% confidence intervals, CI) were estimated. Models were adjusted for maternal age at delivery, parity, neighbourhood income, rurality, health region, and type of health care provider.

Missing neighbourhood income was merged with the lowest income quintile as these neighbourhoods are mostly marginalized.

We performed a sensitivity analysis excluding infants eligible for immunizations or well-child visits prior to their exposure period, restricting the analysis to infants who were a maximum of 2 months old at the start of the pandemic, and children a maximum of 12 months old at the start of the pandemic. Please see Supplementary Tables 3, 4 and 5 for sensitivity analysis results and definitions. In addition, to explore if there were differences in Canada’s largest metropolitan area, we performed a sensitivity analysis utilizing a variable that identified infants and children living in the Greater Toronto Area (GTA). Please see Supplementary Tables 6, 7, 8 and 9 for results.

All statistical analyses were done in SAS ® version 9.4 (SAS Institute, Cary, NC).

## Results

This study included 259,916 infants in Ontario and 33,994 in Manitoba (after exclusions), and 267,469 children in Ontario and 33,001 in Manitoba (after exclusions) (Figure 2). In Manitoba, maternal age was younger, with a greater proportion having had previous pregnancies, and living rurally (Table 1). In both provinces, a high proportion of individuals had a primary care provider, although in Manitoba, children between the ages of 12 and 24 months were least likely to have a primary care provider assigned, especially if exposed to the pandemic.

**Table 1:** Baseline characteristics of the infant and child cohort exposed and unexposed to the pandemic in Ontario and Manitoba, Canada. All data shown as a number (%) unless otherwise indicated

	Ontario				Manitoba			
	Infants < 12 months old		Children 12–24 months old		Infants < 12 months old		Children 12–24 months old	
	Exposed N= 125,000	Unexposed N=134,916	Exposed N= 131,529	Unexposed N=135,940	Exposed N=16,789	Unexposed N=17,205	Exposed N=16,623	Unexposed N=16,378
Maternal characteristics								
Age								
Mean (SD)	31.4 (5.0)	31.1 (5.1)	31.2 (5.1)	30.9 (5.2)	29.8 (5.6)	29.3 (5.6)	29.5 (5.5)	29.1 (5.6)
<20	*1444-1448 (1.2)	*2051-2055 (1.5)	*1744-1748 (1.3)	*2388-2392 (1.8)	695 (4.1)	775 (4.5)	709 (4.3)	817 (5.0)
20-34	89,985 (72.0)	98,596 (73.1)	95,334 (72.5)	100,489 (73.9)	12,644 (75.3)	13,300 (77.3)	12,836 (77.2)	12,709 (77.6)
35+	33,566 (26.9)	34,264 (25.4)	34,446 (26.2)	33,058 (24.3)	3450 (20.5)	3130 (18.2)	3078 (18.5)	2851 (17.4)
Missing	*1-5 (0.0)	*1-5 (0.0)	*1-5 (0.0)	*1-5 (0.0)	0 (0.0)	0 (0.0)	0 (0.0)	0 (0.0)
Parity								
Median (IQR)	1 (0-1)	1 (0-1)	1 (0-1)	1 (0-1)	1 (0-2)	1 (0-2)	1 (0-2)	1 (0-2)
0	57,343 (45.9)	58,954 (43.7)	58,344 (44.4)	59,653 (43.9)	6043 (36.0)	6265 (36.4)	5907 (35.5)	5864 (35.8)
1+	67,647 (54.1)	75,946 (56.3)	73,175 (55.6)	76,280 (56.1)	10,640 (63.4)	10,830 (62.9)	10,601 (63.8)	10,397 (63.5)
Missing	10 (0.0)	16 (0.0)	10 (0.0)	7 (0.0)	106 (0.6)	110 (0.6)	115 (0.7)	117 (0.7)
Rural								
Yes	11,604 (9.3)	13,145 (9.7)	12,509 (9.5)	13,235 (9.7)	7619 (45.4)	7574 (44.0)	7563 (45.5)	7391 (45.1)
Neighbourhood income quintile								
Q1 (lowest)	26,397 (21.1)	29,497 (21.9)	28,482 (21.7)	30,224 (22.2)	4337 (25.8)	4471 (26.0)	4145 (24.9)	4099 (25.0)
Q2	25,076 (20.1)	26,957 (20.0)	26,255 (20.0)	27,049 (19.9)	3646 (21.7)	3717 (21.6)	3614 (21.7)	3482 (21.3)
Q3	26,879 (21.5)	28,309 (21.0)	27,994 (21.3)	28,381 (20.9)	3075 (18.3)	3179 (18.5)	3038 (18.3)	2945 (18.0)
Q4	25,778 (20.6)	27,811 (20.6)	27,082 (20.6)	27,847 (20.5)	3087 (18.4)	3217 (18.7)	3114 (18.7)	3055 (18.7)
Q5 (highest)	20,870 (16.7)	22,342 (16.6)	21,716 (16.5)	22,439 (16.5)	2626 (15.6)	2595 (15.1)	2655 (16.0)	2727 (16.7)
Ontario Transitional Health Regions								
Central	42,230 (33.8)	44,729 (33.2)	43,661 (33.2)	45,419 (33.4)				
East	29,879 (23.9)	32,166 (23.8)	31,510 (24.0)	32,612 (24.0)				
North	5844 (4.7)	7317 (5.4)	6686 (5.1)	7543 (5.5)				
Toronto	11,676 (9.3)	12,438 (9.2)	12,061 (9.2)	12,346 (9.1)				
West	35,343 (28.3)	38,224 (28.3)	37,565 (28.6)	37,982 (27.9)				
Missing data	28 (0.0)	42 (0.0)	46 (0.0)	38 (0.0)				
Manitoba Health Regions								
Interlake Eastern					1465 (8.7)	1476 (8.6)	1567 (9.4)	1520 (9.3)
Northern					1748 (10.4)	1666 (9.7)	1688 (10.2)	1678 (10.2)
Southern					3079 (18.3)	3089 (18.0)	3022 (18.2)	2947 (18.0)
Prairie Mountain					2069 (12.3)	2110 (12.3)	2048 (12.3)	2013 (12.3)
Winnipeg					8428 (50.2)	8864 (51.5)	8298 (49.9)	8220 (50.2)
Primary care provider (yes)	118,544 (94.8)	130,126 (96.4)	126,712 (96.3)	130,907 (96.3)	15,432 (91.9)	16,208 (94.2)	13,497 (81.2)	14,271 (87.1)
Child health provider								
Family physician/General practitioner	81,901 (65.5)	92,060 (68.2)	89,552 (68.1)	92,407 (68.0)	7720 (46.0)	8495 (49.4)	6486 (39.0)	7513 (45.9)
Pediatrician	36,643 (29.3)	38,066 (28.2)	37,160 (28.3)	38,500 (28.3)	1357 (8.1)	997 (5.8)	3126 (18.8)	2107 (12.9)
Primary care nurse					7297 (43.5)	7282 (42.3)	6644 (40.0)	6379 (38.9)
Continuity of care^1^								
Yes	84,394 (67.5)	78,355 (58.1)	78,303 (59.5)	79,595 (58.6)	10,534 (62.7)	9983 (58.0)	9286 (55.9)	8021 (49.0)

SD = standard deviation; IQR = inter quartile range

^*^Suppression of data due to cell sizes less than 6, to ensure no risk of re-identification of patients per institutional policy.

^1^Continuity of care was defined by having ≥76% of visits to the assigned primary care provider

**Fig. 3:**
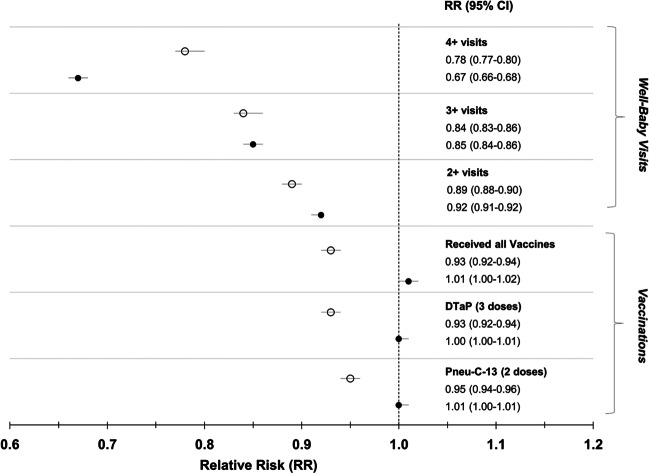
Receipt of vaccinations and well-child visits in Ontario, and Manitoba, comparing infants less than 12 months of age post-pandemic to infants pre-pandemic. The black circles represent Ontario, the white circles represent Manitoba. The Poisson model was adjusted for maternal age at delivery, parity, neighbourhood income, rurality, health region, and health care provider. Received all vaccinations refers to having received 3 doses of DTaP and 2 doses of Pneu-C-13 for infants < 12 months. DTaP = diphtheria, tetanus, pertussis, polio, and haemophilus influenzae type B, Pneu-C-13 = pneumococcal conjugate 13

**Fig. 4:**
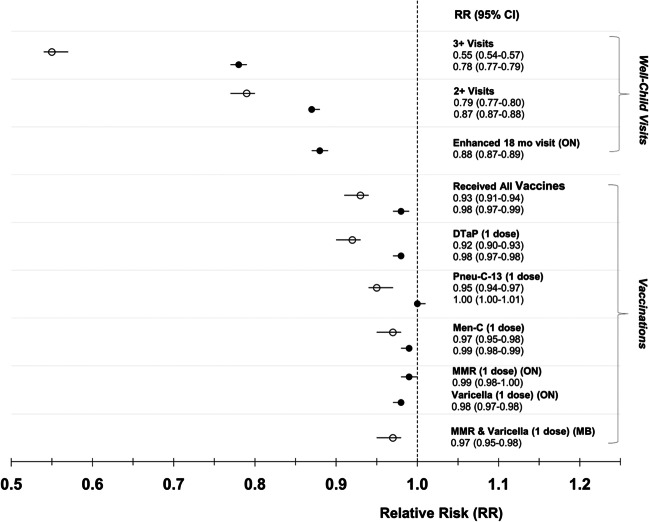
Receipt of vaccinations and well-child visits in Ontario and Manitoba, comparing children between 12 and 24 months old post-pandemic to children pre-pandemic. The black circles represent Ontario, the white circles represent Manitoba. The Poisson model was adjusted for maternal age at delivery, parity, neighbourhood income, rurality, health region, and health care provider. In Ontario, received all vaccinations refers to having received 1 dose each of DTaP, Pneu-C-13, Men-C-C, MMR, and Varicella between the ages of 12 and 24 months. In Manitoba, received all vaccinations refers to having received 1 dose each of DTaP, Pneu-C-13, Men-C-C, MMR, and Varicella between the ages of 12 and 24 months. DTaP = diphtheria, tetanus, pertussis, polio, and haemophilus influenzae type B; Pneu-C-13 = pneumococcal conjugate 13; Men-C-C = meningococcal type C; MMR = measles, mumps and rubella; MMR-V = measles, mumps, and rubella and varicella

Figures 3 and 4 illustrate relative risks and 95% confidence intervals for vaccinations and well-child visit outcomes for the infant and child cohorts, respectively.

### Vaccinations

In Ontario, according to billing data there was an increase of 0.9% in receipt of all vaccinations in children 0-12 months of age during the pandemic compared to prior [62.5% (N=78,175) vs 61.6% (N=83,068), aRR 1.01 (95% CI 1.00-1.02)] (Table 2, Figure 3). In Manitoba, there was a decline of 5.7% [73.5% (N=12,343) vs 79.2% (N=13,621), aRR 0.93 (95% CI 0.92-0.94)] (Figure 3). The proportion of infants who received the recommended number of doses of the individual vaccines was similar to the overall proportion who received all vaccines for both provinces (Table 2).

**Table 2:** Comparison of immunizations and well-child visits for infants younger than 12 months old who were exposed or unexposed to the pandemic. All data shown as a number (%) unless otherwise indicated

	Ontario				Manitoba			
	Exposed N= 125,000		Unexposed N=134,916		Exposed N=16,789		Unexposed N=17,205	
Vaccination outcomes								
*Primary outcome*								
Received all vaccines^1^	78,175 (62.5)		83,068 (61.6)		12,343 (73.5)		13,621 (79.2)	
*Secondary outcome*								
Number of DTaP-IPV-HiB received [Mean (SD)|Median (IQR)]	2.3 (1.1)	3 (2-3)	2.3 (1.1)	3 (2-3)	2.5 (1.0)	3 (2-3)	2.6 (0.9)	3 (3-3)
0	17,764 (14.2)		21,512 (15.9)		1684 (10.0)		1268 (7.4)	
1	7956 (6.4)		7651 (5.7)		957 (5.7)		682 (4.0)	
2	17,918 (14.3)		18,921 (14.0)		1691 (10.1)		1516 (8.8)	
3 or more	81,362 (65.1)		86,832 (64.4)		12,457 (74.2)		13,737 (79.8)	
Number of Pneu-C-13 received [Mean (SD)|Median (IQR)]	1.58 (0.82)	2 (1-2)	1.55(0.84)	2 (1-2)	1.83 (0.75)	2 (2-2)	1.94 (0.71)	2 (2-2)
0	20,709 (16.6)		24,861 (18.4)		1734 (10.3)		1324 (7.7)	
1	16,330 (13.1)		16,262 (12.1)		1214 (7.2)		909 (5.3)	
2 or more	87,961 (70.4)		93,793 (69.5)		13,841 (82.5)		14,972 (87.0)	
Well-child visit outcomes								
*Primary outcome*								
Received 4 visits^2^	41,247 (33.0)		65,810 (48.8)		9235 (55.0)		12,168 (70.7)	
*Secondary outcome*								
Number of visits received [Mean (SD)|Median (IQR)]	2.63 (1.8)	3 (1-4)	3.18 (1.9)	3 (2-5)	3.71 (2.65)	4 (2-5)	5.14 (3.3)	5 (3-7)
0	22,415 (17.9)		19,492 (14.4)		2514 (15.0)		1425 (8.3)	
1	13,306 (10.6)		10,907 (8.1)		1602 (9.50)		1152 (6.70)	
2 or more	89,279 (71.4)		104,517 (77.5)		12,673 (75.5)		14,628 (85.0)	
3 or more	71,960 (57.6)		90,970 (67.4)		11,083 (66.0)		13,544 (78.7)	
4 or more	41,247 (33.0)		65,810 (48.8)		9235 (55.0)		12,168 (70.7)	

^**1**^Infants in Ontario and Manitoba were considered to have received all vaccinations if they received 3 doses of DTaP-IPV-HiB and 2 doses of Pneu-C-13 prior to 12 months of age.

^2^Received all recommended well-child visits prior to 12 months of age.

In Ontario, there was a decline of 0.8% in receipt of all vaccinations in children 12-24 months of age during compared to prior to the pandemic [48.9% (N=64,290) vs 49.7% (N=67,604), aRR 0.98 (95% CI 0.97-0.99)] (Table 3, Figure 4). In Manitoba, this decline was 5.4% [(67.1% (N=11,160) vs 72.5% (N=11,876), aRR 0.93 (95% CI 0.91-0.94)] (Table 3, Figure 4). In both provinces, across pre- and post-pandemic groups, there was a similar rate of receipt of single dose of recommended vaccines.

**Table 3:** Comparison of immunizations and well-child visits of children between the ages of 12 and 24 months. All data shown as a number (%) unless otherwise indicated

	Ontario				Manitoba			
	Exposed N= 131,529		Unexposed N= 135,940		Exposed N=16,623		Unexposed N=16,378	
Vaccination outcomes								
*Primary outcome*								
Received all vaccinations^1^	64,290 (48.9)		67,604 (49.7)		11,160 (67.1)		11,876 (72.5)	
*Secondary outcome*								
Received recommended 1 dose								
DTaP-IPV-HiB	91,035 (69.2)		96,538 (70.8)		12,424 (74.7)		13,532 (82.6)	
Pneu-C-13	96,098 (73.0)		98,952 (72.8)		13,189 (79.3)		13,742 (83.9)	
Men-C-C	95,862 (72.8)		99,972 (73.5)		13,462 (80.9)		13,746 (83.9)	
MMR (ON)	97,696 (74.3)		102,197 (75.2)					
Varicella (ON)	87,584 (66.6)		92,338 (67.9)					
MMRV (MB)					13,842 (83.2)		13,892 (84.8)	
Well-child visit outcomes								
*Primary outcome*								
Received recommended visits^2^								
3 or more	39,758 (30.2)		52,157 (38.4)					
2 or more	72,906 (55.4)		85,845 (63.1)		9143 (55.0)		11,476 (70.1)	
Enhanced 18 month (ON)	72,182 (54.9)		84,395 (62.1)					
*Secondary outcome*								
Number of visits received [Mean (SD)|Median (IQR)]								
Total	1.66 (1.18)	2 (1-3)	1.89 (1.19)	2 (1-3)	1.8 (1.72)	2 (-3)	2.97 (2.68)	2 (1-4)
0	28,720 (21.8)		23,436 (17.2)		4640 (27.9)		2828 (17.3)	
1	29,903 (22.7)		26,659 (19.6)		2840 (17.1)		2074 (12.7)	
2 or more	72,906 (55.4)		85,845 (63.1)		9143 (55.0)		11,476 (70.1)	
3 or more	39,758 (30.2)		52,157 (38.4)					

ON = Ontario, MB = Manitoba

^1^A child was considered to have received all vaccinations if they received 1 dose each of Pneu-C-13, Men-C-C, MMR, Varicella between the ages of 12 and 24 months for Ontario. In Manitoba, a child was considered to have received all vaccinations if they received 1 dose each of Pneu-C-13, Men-C-C, MMR-Varicella between the ages of 12 and 24 months.

^2^Received well-child visits between the ages of 12 and 24 months. In Ontario only, an ‘enhanced 18-month visit’ is recommended, whereas in Manitoba a well-child visit at 18 months occurs. The 18-month visit is included in the total recommended visits.

Supplementary Figures 1 and 2 show the distribution of vaccination doses received by age in months in infants and children. Distributions were similar before and during the pandemic, as well as between provinces.

### Well-child visits

In Ontario, of infants less than 12 months of age post-pandemic, 33.0% (N=41,247) completed at least 4 well-child visits, compared to 48.8% (N=65,810) pre-pandemic [difference = 15.8%, aRR of 0.67 (95% CI 0.66-0.68)] (Table 2, Figure 3). Of those, 17.9% (N=22,415) of infants did not receive any well-child visits compared to 14.4% (N=19,492) pre-pandemic. In Manitoba, 55.0% (N=9235) of infants less than 12 months of age post-pandemic had received 4 or more well-child visits, compared to 70.7% (N=12,168) pre-pandemic [difference = 15.7%, aRR 0.78 (95% CI 0.77-0.80)] (Figure 3). Similar to Ontario, more post-pandemic infants did not have any well-child visits (15%, N=2514) compared to pre-pandemic (8.3%, N=1425).

Children in Ontario post-pandemic were less likely to have received at least 3 well-child visits between 12 and 24 months of age compared to those pre-pandemic (30.2% [N=39,758] vs 38.4% [N=52,157], difference = 8.2%) corresponding to an aRR 0.78 (95% CI 0.77-0.79) (Table 3, Figure 4). Of those post-pandemic, 54.9% (N=72,182) received the enhanced 18-month well-child visit compared to 62.1% (N=84,395) pre-pandemic aRR 0.88 (95% CI 0.87-0.89) (Table 3, Figure 4). In Manitoba, 55.0% (N=9143) of children post-pandemic had received at least 2 well-child visits as recommended in this province between 12 and 24 months of age, compared to 70.1% (N=11,476) pre-pandemic, a decline of 15.1% and aRR 0.79 (95% CI 0.77-0.80) (Table 3, Figure 4).

Sensitivity analyses revealed similar results and there were no clinically important differences between the main and sensitivity analyses results (Supplementary Tables 3, 4, 5, 6, 7, 8 and 9).

## Discussion

In this large population-based study of infants and children across two provinces in a single payer healthcare system, we observed a modest reduction in completion of vaccine series during the COVID-19 pandemic up to September 2021. In contrast, there was a large reduction in completion of recommended well-child visits in both provinces (an absolute difference of 8-15% in Ontario and 15-16% in Manitoba). These findings are important to understand the impact of the pandemic on preventive care delivery, which in turn can inform a tailored response to catch up on missed preventive opportunities.

The change in vaccination rates during the pandemic compared to pre-pandemic was a 0.8% decline in children and a 0.9% increase in infants in Ontario based upon billing data, whereas it was a 5.4-5.7% decline in Manitoba. This interprovincial difference was not anticipated. In Manitoba, public health nurses contribute substantially to rates of childhood immunization, and also perform well-baby visits, where they are responsible for 99.6% of childhood vaccinations in rural Manitoba (Hilderman et al., 2011). During the pandemic, public health nurses were reassigned to case and contact management of COVID-19 (Daly, 2022). About 40% of Manitobans live in non-metropolitan areas, compared to 15% in Ontario. Other factors affecting a lag in catch-up vaccinations could include enforcement of stay-at-home orders, caregiver perception that vaccinations are non-essential, fears of contracting COVID-19 in a healthcare facility, and barriers to accessing well-child visits (Piché-Renaud et al., 2021). Manitoba issued extensive public health restrictions until May 2020 and re-instituted them in August 2020 (Blake-Cameron et al., 2021). First Nations communities in Manitoba, often rural, continued to implement restrictions beyond the provincial mandates. Gradual restrictions in Ontario occurred starting in March 2020 with gradual re-opening by region in May 2020. It is unclear how timing or severity of restrictions between the provinces could have affected access to or uptake of routine childhood vaccination (Blake-Cameron et al., 2021). In addition, Ontario issued personal protective equipment (PPE) at no cost to community family physicians by August 2020, while physicians in Manitoba voiced concerns over the availability of PPE (CPSM, 2020). Both Ontario and Manitoba had public health or expert roundtables call for maintaining routine vaccination in children, resulting in targeted vaccine catch-up programs (Allan et al., 2021; NACI, 2020). Given these dynamic changes during the pandemic, the strength of our study is that it measures the receipt of vaccinations and well-baby visits by September 2021 providing a catch-up period after the period of strict public health restrictions.

Across North America, incomplete and delayed immunization for children under 24 months of age increased in 2020 with some indications for recovery late in 2020 (DeSilva et al., 2022; Dong et al., 2022; Ji et al., 2022; Kujawski et al., 2022; Piché-Renaud et al., 2021; Lee et al., 2022; Saini et al., 2017). This raised concerns over how small changes in vaccine coverage have potentially increased the population risk for vaccine-preventable diseases (Payne, 2022; Piché-Renaud et al., 2021; Sell et al., 2021). Overall, vaccinations after 12 months of age decreased to a greater extent than those prior to 12 months of age (Kiely et al., 2021; Saini et al., 2017). Vaccination coverage (doses of vaccine administered) showed recovery in Quebec with vaccination coverage for MMR below 2019 rates by 2-3% in November 2020 (Kiely et al., 2021). In Alberta, MMR coverage declined by 9.9% in April 2020 compared to 2019, but showed recovery by July 2020 (MacDonald et al., 2022). Rates of immunization uptake vary by parental concerns about visiting health facilities, socioeconomic status, staff shortages, and clinic model (Dong et al., 2022; Ji et al., 2022; Sell et al., 2021). A large cross-sectional study in the United States using commercial healthcare claims databases reported rates of vaccination and well-child visits and showed a persistent decline of 14.3% for the MMR vaccination for 12-month-olds up to May 2021 (Kujawski et al., 2022). Using data from two large provinces, and extending the study period beyond the intensive pandemic measures, we show a modest gap in vaccination, particularly in Ontario, suggesting catch-up mechanisms and decreased pandemic restrictions increased the number of children receiving vaccinations.

Well-child visits are considered the cornerstone of primary care for child health, providing essential screening and opportunity for provision of preventive services and other essential clinical services (Fischer et al., 1984). Delays in providing such essential care causes concerns for delays to needed treatments, particularly developmental interventions. We found a substantial decline in well-child visits in infants and children beyond the first year of the pandemic. Our results are similar to the large cross-sectional study in the USA which also showed a well-child visit decline of 14.2% for children 0-2 years old by January 2021 compared to 2018-2019 (Kujawski et al., 2022). Our results are in keeping with findings of a study in Toronto, Ontario, Canada showing well-child visits reduced by 16% in the first year of the pandemic (Stephenson et al., 2021). However, studies on different health systems or populations have shown inconsistent results, with one study in the USA showing relative stability in the well-child visits below the age of 12 months in a Midwest health care system; and similarly in surveys of parents and practitioners, illustrating the importance of age, race, ethnicity, healthcare model and health insurance considerations when considering the disparities caused by the pandemic with respect to health service use (Salas et al., 2022; Teasdale et al., 2022, Piché-Renaud et al., 2021; DeSilva et al., 2022).

Persistent suboptimal well-child visits rates, and a lower vaccination rate in Manitoba, highlight a need for catch-up or a shift in preventive care provision, such as leveraging sick visits for vaccinations and developmental surveillance (DeSilva et al., 2022).

### Limitations of the study

Our study has several strengths related to the use of administrative data in two large provinces in Canada with varying pandemic restrictions. However, our study has several limitations. In Ontario, there is no established comprehensive and linkable immunization registry and the use of vaccine-specific immunization billing codes has specificity ranging from 81% to 92% and sensitivity ranging from 70% to 83% (Schwartz et al., 2015). Vaccination coverage rates for at least 4 doses of DTaP by 24 months was estimated to be 75.7% in Ontario and 67.5% in Manitoba by the Canadian National Childhood Immunization Survey (NCIS) in 2017 (Public Health Agency of Canada, 2020). In our study, Manitoba estimates of vaccination rates were consistent with the estimates by the NCIS. The Ontario immunization rates reported in our study are likely underestimates as vaccine data in this province are dependent on completeness and accuracy of physician billing (Schwartz et al., 2015, Saunders et al., 2021). Children receiving vaccines without associated billing in Ontario, such as through nurse practitioners and salaried physicians, or nurses associated with physicians who did not bill for the procedure, would not have been included in the Ontario data. However we do not anticipate there would be differences in billing practices prior to the pandemic compared to during the pandemic causing a bias across the exposure. In Manitoba, although vaccinations by public health nurses were captured, well-baby visits by the same public health nurses were not, potentially causing a larger underestimation of the visit rate measured during the pandemic.

## Conclusion

Through the first 18 months of the pandemic, we found that routine childhood vaccines were delivered to infants and children at close to pre-pandemic rates in Ontario and Manitoba. In contrast, we document a significant decrease in well-child visits for infants and children, indicating that developmental surveillance catch-up for infants and children is crucial.

## Contributions to knowledge

What does this study add to existing knowledge?

- In two provinces in Canada, the reduction in vaccine uptake was modest beyond the first year of the pandemic, however well-child visits were significantly reduced within the same time period.

What are the key implications for public health interventions, practice or policy?

- Differences in vaccination rates changes pre and during the pandemic demonstrate how routine vaccinations were disrupted differently in a mixed rural/urban compared to a predominantly urban province.
- A decrease in well-child visits underscores the need for catch-up visits for developmental surveillance.

## Supplementary Information

Below is the link to the electronic supplementary material.

Supplementary file1 (PDF 694 KB)

Supplementary file2 (PDF 254 KB)

## Data Availability

Parts of this material are based on data and information compiled and provided by MOH and the Canadian Institute for Health Information, current to September 30, 2021. Geographical data are adapted from Statistics Canada, Postal Code Conversion File +2011 (Version 6D) and 2016 (Version 7B). The analyses, conclusions, opinions and statements expressed herein are solely those of the authors and do not reflect those of the funding or data sources; no endorsement is intended nor should be inferred.

## Code availability

The dataset from this study is held securely in coded form at ICES and the Manitoba Centre for Health Policy (MCHP). While data sharing agreements prohibit ICES and MCHP from making the dataset publicly available, access may be granted to those who meet pre-specified criteria for confidential access, available at www.ices.on.ca/DAS and https://www.umanitoba.ca/faculties/health_sciences/medicine/units/chs/departmental_units/mchp/resources/access.html. The full dataset creation plan and underlying analytic code are available from the authors upon request, understanding that the computer programs may rely upon coding templates or macros that are unique to ICES and MCHP.

## References

[CR1] Allan, K., Piché-Renaud, P.-P., Bartoszko, J., Bucci, L. M., Kwong, J., Morris, S., Pernica, J., & Fadel, S. (2021). Maintaining Immunizations for School-Age Children During COVID-19. https://www.dlsph.utoronto.ca/wp-content/uploads/2021/09/Maintaining-Immunizations-for-School-Age-Children-During-COVID-19-Report-1.pdf

[CR2] Blake-Cameron, E., Breton, C., Sim, P., Tatlow, H., Hale, T., Wood, A., & Tyson, K. (2021). Variation in the Canadian provincial and territorial responses to COVID-19. *BSG Working Paper Series* (BSG-WP-2021/039).

[CR3] Canadian Task Force on Preventive Health Care. (2016). Recommendations on screening for developmental delay. *Canadian Medical Association Journal, 188*(8), 579–587. https://www.cmaj.ca/content/188/8/579 10.1503/cmaj.151437 PMC4868607 27026672

[CR4] Causey, K., Fullman, N., Sorensen, R. J. D., Galles, N. C., Zheng, P., Aravkin, A., Danovaro-Holliday, M. C., Martinez-Piedra, R., Sodha, S. V., Velandia-González, M. P., Gacic-Dobo, M., Castro, E., He, J., Schipp, M., Deen, A., Hay, S. I., Lim, S. S., & Mosser, J. F. (2021). Estimating global and regional disruptions to routine childhood vaccine coverage during the COVID-19 pandemic in 2020: a modelling study. *The Lancet, 398*(10299), 522–534. 10.1016/S0140-6736(21)01337-4 10.1016/S0140-6736(21)01337-4 PMC8285122 34273292

[CR5] Chiem, A., Olaoye, F., Quinn, R., & Saini, V. (2022). Reasons and suggestions for improving low immunization uptake among children living in low socioeconomic status communities in Northern Alberta, Canada - A qualitative study. *Vaccine*. (1873–2518 (Electronic)). 10.1016/j.vaccine.2022.06.004 35701329

[CR6] Daly, M. (2022). *Public Health Nurses a vital part of the fight against COVID-19*. Retrieved from https://wrha.mb.ca/2021/05/10/public-health-nurses-a-vital-part-of-the-fight-against-covid-19/

[CR7] DeSilva, M. B., Haapala, J., Vazquez-Benitez, G., Daley, M. F., Nordin, J. D., Klein, N. P., & Kharbanda, E. O. (2022). Association of the COVID-19 pandemic with routine childhood vaccination rates and proportion up to date with vaccinations across 8 US health systems in the vaccine safety datalink. (2168–6211 (Electronic)). 10.1001/jamapediatrics.2021.4251 PMC8498937 34617975

[CR8] Doctors Manitoba. (2021, April 8). Well Baby Care. https://doctorsmanitoba.ca/managing-your-practice/remuneration/billing-fees/visits/well-baby-care

[CR9] Dong, A., Meaney, C., Sandhu, G., De Oliveira, N., Singh, S., Morson, N., & Forte, M. (2022). Routine childhood vaccination rates in an academic family health team before and during the first wave of the COVID-19 pandemic: a pre-post analysis of a retrospective chart review. (2291–0026 (Electronic)). 10.9778/cmajo.20210084 PMC8920592 35078822

[CR10] Fischer, P. J., Strobino, D. M., & Pinckney, C. A. (1984). Utilization of child health clinics following introduction of a copayment. *American Journal of Public Health, 74*(12), 1401–1403. 10.2105/ajph.74.12.1401 PMC1652676 6507695

[CR11] Glazier, R., & Rayner, J., & Kopp, A. (2015). *Examining Community Health Centres According to Geography and Priority Populations Served 2011/12 to 2012/13* (p. 2015). Institute for Clinical Evaluative Sciences.

[CR12] Hilderman, T., Katz, A., Derksen, S., McGowan, K., Chateau, D., Kurbis, C., & Reimer, J. (2011). *Manitoba Immunization Study*. http://mchp-appserv.cpe.umanitoba.ca/deliverable.php?referencePaperID=76539

[CR13] Ji, C., Piché-Renaud, P. P., Apajee, J., Stephenson, E., Forte, M., Friedman, J. N., & Tu, K. (2022). Impact of the COVID-19 pandemic on routine immunization coverage in children under 2 years old in Ontario, Canada: A retrospective cohort study. (1873–2518 (Electronic)). 10.1016/j.vaccine.2022.02.008 PMC8824235 35164987

[CR14] Kiely, M., Mansour, T., Brousseau, N. A.-O., Rafferty, E., Paudel, Y. A.-O., Sadarangani, M., & MacDonald, S. A.-O. (2021). COVID-19 pandemic impact on childhood vaccination coverage in Quebec, Canada. (2164–554X (Electronic)). 10.1080/21645515.2021.2007707 PMC9553134 34920686

[CR15] Kujawski S, Yao L, Wang H, Carias C, Chen Y (2022). Impact of the COVID-19 pandemic on pediatric and adolescent vaccinations and well child visits in the United States: A database analysis. Vaccine.

[CR16] Lee, D. I. D., Vanderhout, S., Aglipay, M., Birken, C. S., Morris, S. K., Piché-Renaud, P. P., Keown-Stoneman, C. D. G., & Maguire, J. L. (2022). Delay in childhood vaccinations during the COVID-19 pandemic. *Can J Public Health, 113*(1):126–134. 10.17269/s41997-021-00601-9 10.17269/s41997-021-00601-9 PMC8773389 35060107

[CR17] MacDonald, S. E., Paudel, Y. R., Kiely, M., Rafferty, E., Sadarangani, M., Robinson, J. L., Driedger, S. M., & Svenson, L. W. (2022). Impact of the COVID-19 pandemic on vaccine coverage for early childhood vaccines in Alberta, Canada: a population-based retrospective cohort study. *BMJ Open, 12*, e055968. 10.1136/bmjopen-2021-055968 10.1136/bmjopen-2021-055968 PMC8795926 35078849

[CR18] National Advisory Committee on Immunization (NACI). (2020, May 13). Interim guidance on continuity of immunization programs during the COVID-19 pandemic. National Advisory Committee on Immunization (NACI). https://www.canada.ca/en/public-health/services/immunization/national-advisory-committee-on-immunization-naci/interim-guidance-immunization-programs-during-covid-19-pandemic.html

[CR19] Payne, E. (2022, September 20). Dropping childhood immunization rates are putting children at risk: Dr. Vera Etches. Ottawa Citizen. https://ottawacitizen.com/news/local-news/dropping-childhood-immunization-rates-are-putting-children-at-risk-dr-vera-etches

[CR20] Piché-Renaud, P. P., Ji, C., Farrar, D. S., Friedman, J. N., Science, M., Kitai, I., Morris, S. K. (2021). Impact of the COVID-19 pandemic on the provision of routine childhood immunizations in Ontario, Canada. (1873–2518 (Electronic)). 10.1016/j.vaccine.2021.05.094 PMC9756828 34108076

[CR21] Public Health Agency of Canada. (2020). Vaccine Coverage in Canadian Children: Results from the 2017 Childhood National Immunization Coverage Survey (cNICS). https://www.canada.ca/en/public-health/services/publications/healthy-living/2017-vaccine-uptake-canadian-children-survey.html

[CR22] Rigby, J. (2022). Why has polio been found in London, New York and Jerusalem, and how dangerous is it? *CTV News.*

[CR23] Rourke, J. T., & Rourke, L. L. (1985). Well baby visits: screening and health promotion. *Canadian Family Physician, 31*, 997–1002. PMC2327796 21274145

[CR24] Saini, V., MacDonald, S. E., McNeil, D. A., McDonald, S. W., Kellner, J. D., Edwards, S. A., Stagg, V., & Tough, S. (2017). Timeliness and completeness of routine childhood vaccinations in children by two years of age in Alberta, Canada. *Can J Public Health, 108*(2), e124–e128. 10.17269/CJPH.108.5885 PMC6972325 28621647

[CR25] Salas, J., Hinyard, L., Cappellari, A., Sniffen, K., Jacobs, C., Karius, N., Grucza, R. A., & Scherrer, J. F. (2022). Infant, pediatric and adult well visit trends before and during the COVID-19 pandemic: a retrospective cohort study. *BMC Health Services Research, 22*(1), 328. 10.1186/s12913-022-07719-7 10.1186/s12913-022-07719-7 PMC8916698 35277169

[CR26] Saunders N, Guttmann A, Brownell M, Cohen E, Fu L, Guan J, Stukel T (2021). Pediatric primary care in Ontario and Manitoba after the onset of the COVID-19 pandemic: a population-based study. CMAJ Open.

[CR27] Schwartz, K. L., Tu, K., Wing, L., Campitelli, M. A., Crowcroft, N. S., Deeks, S. L., Wilson, S. E., Wilson, K., Gemmill, I., & Kwong, J. C. (2015). Validation of infant immunization billing codes in administrative data. *Hum Vaccin Immunother, 11*(7):1840–1847. 10.1080/21645515.2015.1043499 10.1080/21645515.2015.1043499 PMC4514409 26075651

[CR28] Sell H, Assi A, Driedger SM, Dube E, Gagneur A, Meyer SB, MacDonald S (2021). Continuity of routine immunization programs in Canada during the COVID-19 pandemic. Vaccine..

[CR29] Stephenson E, Butt D, Gronsbell J, Ji C, O’Neill B, Crampton N (2021). Changes in the top 25 reasons for primary care visits during the COVID-19 pandemic in a high-COVID region of Canada. PLoS One.

[CR30] Teasdale, C. A., Borrell, L. N., Shen, Y., Kimball, S., Zimba, R., Kulkarni, S., Rane, M., Rinke, M. L., Fleary, S. A., & Nash, D. (2022). Missed routine pediatric care and vaccinations in US children during the first year of the COVID-19 pandemic. *Preventive Medicine, 158*, 107025. 10.1016/j.ypmed.2022.107025 10.1016/j.ypmed.2022.107025 PMC8933962 35318030

[CR31] The College of Physicians & Surgeons of Manitoba (CPSM). (2020). Personal Protective Equipment during COVID-19. https://cpsm.mb.ca/assets/COVID19/PPE%202020%2004%2006.pdf

[CR32] Walton, S., Cortina-Borja, M., Dezateux, C., Griffiths, L. J., Tingay, K., Akbari, A., Bedford, H. (2022). Linking cohort data and Welsh routine health records to investigate children at risk of delayed primary vaccination. (1873–2518 (Electronic)). 10.1016/j.vaccine.2022.06.080 PMC10499753 35842339

[CR33] Williams, R., Clinton, J., & Biscaro, A. (2008). Ontario and the enhanced 18-month well-baby visit: Trying new approaches. *Paediatrics & Child Health, 13*(10), 850–856. 10.1093/pch/13.10.850 10.1093/pch/13.10.850 PMC2603505 19436551

[CR34] World Health Organization (WHO). (2020). Guiding principles for immunization activities during the COVID-19 pandemic: interim guidance. World Health Organization. https://apps.who.int/iris/bitstream/handle/10665/331590/WHO-2019-nCoV-immunization_services-2020.1-eng.pdf?sequence=1&isAllowed=y

