# Supplementary material for: Gaps in childhood immunizations and preventive care visits during the COVID-19 pandemic: a population-based cohort study of children in Ontario and Manitoba, Canada, 2016–2021
Source: Can J Public Health. 2023 Jul 13;114(5):774–86. doi: 10.17269/s41997-023-00797-y (PMC10484833; doi:10.17269/s41997-023-00797-y)
Supplement: Supplementary file 1 — Supplementary file1 (PDF 694 KB) [file 41997_2023_797_MOESM1_ESM.pdf]

Gaps in childhood immunizations and preventative care visits during the COVID-19 pandemic – A population-based cohort study of children in Ontario and Manitoba, Canada, 2016 - 2021

Canadian Journal of Public Health

Andrea Evans[1-3], Alyson L Mahar[4,5], Bhumika Deb[3], Alexa Boblitz[3], Marni Brownell[4-6], Astrid Guttmann[3,8,-13], Therese Stukel[3], Eyal Cohen[3, 8-13], Joykrishna Sarkar[5], Nkiruka Eze[5], Alan Katz[4-6], Tharani Raveendran[9], Natasha Saunders[3,8-13]

[1]Children's Hospital of Eastern Ontario, Ottawa, Canada;
[2]Department of Pediatrics, University of Ottawa, Ottawa, Canada;
[3]ICES, Toronto, Canada
[4]Department of Community Health Sciences, University of Manitoba, Winnipeg, Canada
[5]Manitoba Centre for Health Policy, Winnipeg, Manitoba
[6]Children's Hospital Research Institute of Manitoba, Winnipeg, Manitoba
[7]Department of Family Medicine University of Manitoba, Winnipeg, Manitoba
[8]The Hospital for Sick Children, Toronto, Canada
[9]Child Health Evaluative Sciences, SickKids Research Institute, Toronto, Canada
[10]Department of Pediatrics, University of Toronto, Toronto, Canada
[11]Institute of Health Policy, Management and Evaluation, The University of Toronto, Toronto, Canada
[12]Temerty Faculty of Medicine, University of Toronto, Toronto, Canada
[13]Edwin S.H. Leong Centre for Healthy Children, University of Toronto, Toronto, Canada

Corresponding author: Natasha Saunders, Natasha.saunders@sickkids.ca,
T: 416-813-7654 x 203076

Supplementary Materials

Table 1. Data sources and associated study variables.

Data Source	Data Elements	Variables	Province
Canadian Institute for Health Information Discharge Abstract Database (DAD)	Record of hospital admission data. Diagnostic codes associated with hospitalizations.	Parity<br>Infant or child Sex	Ontario
Ontario Health Insurance Plan (OHIP)	Billing claims in Ontario.	Well-child Visits<br>Immunizations	Ontario
Registered Person's Database (RPDB)	The RPDB provides basic demographic information (age, sex, location of residence, date of birth) for those issued an Ontario health insurance number.	Maternal Age Neighbourhood income quintile<br>Ontario Transitional Health Regions	Ontario
2016 Canadian Census	The most recent Census of Canada which takes place every five years in Canada providing population and dwelling counts, demographic and other socioeconomic characteristics.	Rurality<br>Neighbourhood income quintile	Ontario, Manitoba
Hospital Discharge Abstracts	Record of hospital admission data. Diagnostic codes associated with hospitalizations.	Parity	Manitoba
Manitoba Health Insurance Registry	Tracks addresses and dates of birth, death and insurance	Infant or child Sex	Manitoba

	coverage for all insured persons.	Residential postal code, used for neighbourhood income quintile and health regions<br>Date of birth<br>Maternal age	
Pubic Health Information Management System (PHIMS)	Population-based province-wise public health record including childhood vaccinations administered in Manitoba 20xx-20xx	Vaccine type, administration date	Manitoba
Manitoba Medical Services	Billing claims	Well-child visits	Manitoba

Table 2: List of primary care billing codes from Ontario and Manitoba

**ONTARIO**	
**Fee Code (OHIP)**	**Description**
Well-child Visits	
A002	18 MONTH WELL BABY CHECK -GP/FP
A268	18 MONTH WELL BABY CHECK - PAEDS
A007	INTERMED.ASSESS/WELL BABY CARE-F.P./G.P./PAED. With diagnostic code 916 or 917
A262	LEVEL 2 PEDIATRIC ASSESSMENT with diagnostic code 916 or 917
Immunizations	
G840	DTaPIPV-Diphtheria, Tetanus, acellular Pertussis, Inactivated Polio Virus paediatric
G841	DTaPIPVHib-Diphtheria, Tetanus, acellular Pertussis, Inactivated Polio Virus, Haemophilus influenza type b paediatric
G844	MenCC-Meningococcal C Conjugate
G845	MMR-Measles, Mumps, Rubella
G846	Pneu - Pneumococcal Conjugate
G848	Var-Varicella
**MANITOBA**	
**ICD-10 Code**	**Description**

Well-child Visits	
First we identified ambulatory primary care visits using the following inclusions/exclusions from the Medical clams data:<br>1. Keep all records with PREFIX='7'<br>'Exclusion1: In-Patient visits';<br>'Exclusion2: EENT-Optometry, Unknown, Emergency Medicine (MDBLOC=053, 99, 113) visits';<br>'Exclusion3: Emergency room services visits';<br>'Exclusion4: Out of Province claims (The 3rd character in MDBLOC=9)';<br>'Exclusion5: Chiropractic claims (diagnoses start with A, B, or C)';<br>'Exclusion6: Non-Primary care visits (by keeping mdbloc in ('02', '11', '111', '112', '114', '115', '116', '200')) ';<br><br>Then, Well baby visit is defined using only tariff codes ('8734', '8529', '8523').<br>8523: Well Baby Care by a physician other than a Paediatrician or a General Practitioner<br>8529: Regional Intermediate Visit—Regional or Subsequent Visit or Well Baby Care<br>8734: Extended Regional Intermediate Visit—Regional or Subsequent Visit or Well Baby Care—minimum of thirty (30) minutes of patient/physician contact time<br>(Source: https://www.gov.mb.ca/health/documents/physmanual.pdf)	
Immunizations	
List of snomed codes are used to define immunization based on PHIMS data:<br>('7831000087100', '7921000087109', '59999009', '8211000087108', '7901000087103', '7931000087106', '7911000087101', '7881000087101', '8211000087108', '333680004','359068008', '419550004', '386012008', '61153008', '125714002', '448964007', '7661000087108', '125690004', '7901000087103', '125688000', '7811000087105', '7821000087102', '62294009', '108729007', 'MB8908', 'MB8968', 'MB8969', 'SCTMB172', 'SCTMB181')	

Table 3. Sensitivity Analysis **-** Baseline characteristics of the infant and child cohort exposed and unexposed to the pandemic in Ontario and Manitoba, Canada**.** All data shown as a number (%) unless otherwise indicated.

	**Ontario**				**Manitoba**			
	**Infants < 12 months old**		**Children 12 - 24 months old**		**Infants < 12 months old**		**Children 12 – 24 months old**	
	Exposed N= 86,312	Unexposed N=134,959	Exposed N= 72,303	Unexposed N=135,957	Exposed N=9,179	Unexposed N=17,205	Exposed N=9,280	Unexposed N=16,378

**Maternal Characteristics**								
**Age**								
Mean (SD)	31.4 (5.0)	31.1 (5.1)	31.2 (5.0)	30.9 (5.2)	29.7 (5.6)	29.3 (5.6)	29.5 (5.5)	29.1 (5.6)
<20	*970-974	*2,052-2,056	*957-961	*2388-2392	365 (4.0)	775 (4.5)	391 (4.2)	817 (5.0)
20-34	62,011 (71.8)	98,634 (73.1)	52,706 (72.9)	100,501 (73.9)	6,962 (75.8)	13,300 (77.3)	7,175 (77.3)	12,709 (77.6)
35+	23,326 (27.0)	34,268 (25.4)	18,635 (25.8)	33,063 (24.3)	1,852 (20.2)	3,130 (18.2)	1,714 (18.5)	2,851 (17.4)
Missing	*1-5	*1-5	*1-5	*1-5	0 (0.0)	0 (0.0)	0 (0.0)	0 (0.0)
**Parity**								
Median (IQR)	1 (0-1)	1 (0-1)	1 (0-1)	1 (0-1)	1 (0-2)	1 (0-2)	1 (0-2)	1 (0-2)
0	39,741 (46.0)	58,966 (43.7)	32,103 (44.4)	59,656 (43.9)	3,294 (35.9)	6,265 (36.4)	3,287 (35.4)	5,864 (35.8)
1+	46,564 (53.9)	75,977 (56.3)	40,194 (55.6)	76,294 (56.1)	5,828 (63.5)	10,830 (62.9)	5,928 (63.9)	10,397 (63.5)
Missing	7 (0.0)	16 (0.0)	6 (0.0)	7 (0.0)	57 (0.6)	110 (0.6)	65 (0.7)	117 (0.7)
**Rural**								
Yes	8,097 (9.4)	13,162 (9.8)	6,946 (9.6)	13,241 (9.7)	4,199 (45.7)	7,574 (44.0)	4,271 (46.0)	7,391 (45.1)
**Neighbourhood income quintile**								
Q1 (lowest)	18,011 (20.9)	29,511 (21.9)	15,412 (21.3)	30,232 (22.2)	2,367 (25.8)	4,471 (26.0)	2,316 (25.0)	4,099 (25.0)
Q2	17,174 (19.9)	26,966 (20.0)	14,435 (20.0)	27,050 (19.9)	2,008 (21.9)	3,717 (21.6)	1,987 (21.4)	3,482 (21.3)
Q3	18,607 (21.6)	28,317 (21.0)	15,525 (21.5)	28,384 (20.9)	1,668 (18.2)	3,179 (18.5)	1,697 (18.3)	2,945 (18.0)
Q4	17,889 (20.7)	27,821 (20.6)	14,965 (20.7)	27,850 (20.5)	1,691 (18.4)	3,217 (18.7)	1,744 (18.8)	3,055 (18.7)
Q5 (highest)	14,631 (17.0)	22,344 (16.6)	11,966 (16.5)	22,441 (16.5)	1,433 (15.6)	2,595 (15.1)	1,519 (16.4)	2,727 (16.7)
**Ontario Transitional Health Regions**								
Central	29,039 (33.6)	44,738 (33.1)	23,833 (33.0)	45,424 (33.4)				
East	20,520 (23.8)	32,177 (23.8)	17,382 (24.0)	32,614 (24.0)				
North	4,073 (4.7)	7,329 (5.4)	3,700 (5.1)	7,551 (5.6)				
Toronto	8,006 (9.3)	12,438 (9.2)	6,558 (9.1)	12,347 (9.1)				
West	24,659 (28.6)	38,235 (28.3)	20,809 (28.8)	37,983 (27.9)				
Missing Data	15 (0.0)	42 (0.0)	21 (0.0)	38 (0.0)				

**Manitoba Health Regions**								
Interlake Eastern					806 (8.8)	1,476 (8.6)	885 (9.5)	1,520 (9.3)
Northern					944 (10.3)	1,666 (9.7)	926 (10.0)	1,678 (10.2)
Southern					1,714 (18.7)	3,089 (18.0)	1,719 (18.5)	2,947 (18.0)
Prairie Mountain					1,149 (12.5)	2,110 (12.3)	1,168 (12.6)	2,013 (12.3)
Winnipeg					4,566 (49.7)	8,864 (51.5)	4,582 (49.4)	8,220 (50.2)
Missing Data					0 (0.0)	0 (0.0)	0 (0.0)	0 (0.0)
**Primary Care Provider (yes)**	81,533 (94.5)	130,156(96.4)	69,495 (96.1)	130,915 (96.3)	8,409 (91.6)	16,208 (94.2)	7,348 (79.2)	14,271 (87.1)
**Child health provider**								
Family Physician/ General Practitioner	56,142 (65.0)	92,085 (68.2)	49,081 (67.9)	92,414 (68.0)	4,117 (44.9)	8,495 (49.4)	3,498 (37.7)	7,513 (45.9)
Pediatrician	25,391 (29.4)	38,071 (28.2)	20,414 (28.2)	38,501 (28.3)	770 (8.4)	997 (5.8)	1,932 (20.8)	2,107 (12.9)
Primary Care Nurse					4,052 (44.1)	7,282 (42.3)	3,645 (39.3)	6,379 (38.9)
No assigned provider	4,779 (5.5)	4,803 (3.6)	2,808 (3.9)	5,042 (3.7)	240 (2.6)	431 (2.5)	205 (2.2)	379 (2.3)
**Continuity of care[1]**								
Yes	59,563 (69.0)	78,372 (58.1)	43,751 (60.5)	79,600 (58.5)	5,772 (62.9)	9,983 (58.0)	5,338 (57.5)	8,021 (49.0)

SD = standard deviation; IQR = inter quartile range

The sensitivity analysis restricted the infant cohort to infants born after January 1st, 2020 (wherein they were a maximum age of 2 months of age at the start of the COVID-19 pandemic). Similarly, children were restricted to be born after March 1, 2019 (wherein they were a maximum of 12 months old at the start of the COVID-19 pandemic).

* Suppression of data due to cell sizes less than six, to ensure no risk of re-identification of patients per institutional policy.

[1]Continuity of care was defined by having ≥76% of visits to the assigned primary care provider

Table 4. Sensitivity Analysis - Comparison of immunizations and well-child visits for infants younger than 12 months old who were exposed or unexposed to the pandemic. All data shown as a number (%) unless otherwise indicated.

	**Ontario**				**Manitoba**			
	Exposed N= 88,341		Unexposed N=1355,185		Exposed N=9,179		Unexposed N=17,205	
**Vaccination Outcomes**								
Primary Outcome								
**Received all vaccines[1]**	**55,290 (62.6)**		**83,094 (61.5)**		**6,658 (72.5)**		**13,621 (79.2)**	
Secondary Outcome								
Number of DTaP-IPV-HiB received [Mean (SD)|Median (IQR)]	2.3 (1.1)	3 (2-3)	2.3 (1.1)	3 (2-3)	2.5 (1.0)	3 (2-3)	2.6 (0.9)	3 (3-3)
0	13,346 (14.3)		21, 696 (16.0)		1,008 (11.0)		1,268 (7.4)	
1	5,474 (6.1)		7,680 (5.7)		562 (6.1)		682 (4.0)	
2	12,049 (13.6)		18,949(14.0)		896 (9.8)		1,516 (8.8)	
3 or more	557,472 (65.1)		86,860 (64.3)		6,713 (73.2)		13,737 (79.8)	
Number of Pneu-C-13 received [Mean (SD)|Median (IQR)]	1.59 (0.82)	2 (1-2)	1.55(0.84)	2 (1-2)	1.83 (0.77)	2 (2-2)	1.94 (0.71)	2 (2-2)
0	15,338 (17.4)		25,047 (18.5)		1,031 (11.2)		1,324 (7.7)	
1	11,261 (12.7)		16,297 (12.1)		676 (7.4)		909 (5.3)	
2 or more	61,742(69.9)		93,841(69.4)		7,472 (81.4)		14,972 (87.0)	
**Well-child Visit Outcomes**								
Primary Outcome								
**Received 4 recommended visits [2]**	29,064 (32.9)		65,835 (48.7)		4,972 (54.2)		12,168 (70.7)	
Secondary Outcome								
Number of visits received [Mean (SD)|Median (IQR)]	2.6 (1.8)	3 (1-4)	3.17 (1.9)	3 (2-5)	3.59 (2.58)	4 (1-5)	5.14 (3.29)	5 (3-7)
0	16,883 (19.1)		19,652(14.5)		1,468 (16.0)		1,425 (8.3)	
1	9,441 (10.7)		10,939 (8.1)		852 (9.3)		1,152 (6.7)	
2 or more	11,682 (13.2)		13,572(10.0)		854 (9.3)		1,084 (6.3)	
3 or more	21,301 (24.1)		25,187 (18.6)		1,033 (11.3)		1,376 (8.0)	
4 or more	29,064 (32.9)		65,835 (48.8)		4,972 (54.2)		12,168 (70.7)	

The sensitivity analysis restricted the infant cohort to infants born after January 1st, 2020 (wherein they were a maximum age of 2 months of age at the start of the COVID-19 pandemic). Infants who died or lost OHIP eligibility were not included in the analysis.

[1]Infants were considered to have received all vaccinations if they received three doses of DTaP-IPV-HiB two doses of Pneu-C-13, in Ontario and Manitoba prior to 12 months.

[2]Received all recommended well-child visits prior to 12 months of age.

Table 5. Sensitivity Analysis - Comparison of immunizations and well-child visits of children between the ages of 12 to 24 months. All data shown as a number (%) unless otherwise indicated.

	**Ontario**				**Manitoba**			
	Exposed N= 72,815		Unexposed N= 135,063		Exposed N=9,280		Unexposed N=16,378	
**Vaccination Outcomes**								
Primary Outcome								
**Received all vaccinations[1]**	36,159 (49.7)		67,605 (49.7)		6,097 (65.7)		11,876 (72.5)	
Secondary Outcome								
**Number of Doses Received**								
DTaP-IPV-HiB	0.71(0.51)		0.71 (0.52)		0.73 (0.49)		0.83 (0.46)	
Pneu-C-13	0.73(0.47)		0.73 (0.48)		0.78 (0.44)		0.84 (0.43)	
Men-C-C	0.73(0.47)		0.74 (0.47)		0.80 (0.40)		0.84 (0.38)	
MMR (MB)	0.74(0.46)		0.75 (0.47)					
Varicella (MB)	0.67(0.49)		0.68 (0.49)					
MMR-V (ON)					0.80 (0.41)		0.83 (0.39)	
**Well-child Visit Outcomes**								
Primary Outcome								
**Received recommended visits[2]**								
3 visits	22,383 (31.0)		52,156(38.4)		2,012 (21.7)		7,959 (48.6)	
2 visits	17,453 (24.0)		33,689 (24.8)		4,791 (51.6)		11,476 (70.1)	
Enhanced 18 month (ON)[3]	41,079(56.8)		84,395 (62.1)					
Secondary Outcome								
**Number of visits received [Mean (SD)|Median (IQR)]**								
Total	1.65 (1.2)	2 (1-3)	1.88 (1.2)	2 (1-3)	1.59 (1.53)	2 (0-2)	2.97 (2.68)	2 (1-4)
0	16,690 (22.4)		23,558 (17.3)		2,878 (31.0)		2,828 (17.3)	
1	16,289 (22.5)		26,660 (19.6)		1,611 (17.4)		2,074 (12.7)	

2 or more	17,443 (24.0)	33,6889 (24.8)	2,779 (29.9)	3,517 (21.5)
3 or more	22,380 (30.7)	52,157 (38.3)	2,005 (21.6)	7,951 (48.2)

ON = Ontario, MB = Manitoba

Sensitivity analysis restricted children born after March 1, 2019 (wherein they were a maximum of 12 months old at the start of the COVID-19 pandemic).

[1]A child was considered to have received all vaccinations if they received one dose of each Pneu-C-13, Men-C-C, MMR, Varicella between the age of 12 and 24 months for Ontario. In Manitoba, a child was considered to have received all vaccinations if they received one dose of each Pneu-C-13, Men-C-C, MMR-Varicella between the age of 12 and 24 months.

[2]Received well-child visits between the ages of 12 and 24 months.

[3]In Ontario only, an 'enhanced 18 month visit' is recommended, whereas in Manitoba a well-child visit at 18 month occurs.

Table 6. Baseline characteristics of the infants and child cohort exposed and unexposed to the pandemic in the Greater Toronto Area (GTA) in Ontario, Canada. All data shown as a number (%) unless otherwise indicated.

	**Ontario**			
	**Infants (<12 months old)**		**Children (12 – 24 months old)**	
	Exposed N=57,381	Unexposed N=61,009	Exposed N=59,490	Unexposed N=61,667
**Maternal Characteristics**				
Age				
Mean (SD)	32.4 (4.8)	32.2 (4.9)	32.3 (4.8)	32.0 (4.9)
<20	330 (0.6)	403 (0.7)	356 (0.6)	435 (0.7)
20-34	38,119 (66.4)	41,120 (67.4)	39,714 (66.8)	42,190 (68.4)
35+	18,932 (33.0)	19,486 (31.9)	19,420 (32.6)	19,042 (30.9)
Missing	0 (0.0)	0 (0.0)	0 (0.0)	0 (0.0)
**Parity**				
Median (IQR)	1 (0-1)	1 (0-1)	1 (0-1)	1 (0-1)
0	*27,595-27,599 (48.1)	*27,761-27,765 (45.5)	*27,502-27,506 (46.2)	*28,023-28,027 (45.4)
1+	29,781 (51.9)	33,243 (54.5)	31,983 (53.8)	33,639 (54.5)
Missing	*1-5 (0.0)	*1-5 (0.0)	*1-5 (0.0)	*1-5 (0.0)
**Rural**				
Yes	88 (0.2)	99 (0.2)	84 (0.1)	62 (0.1)
**Neighbourhood Income Quintile**				
Q1 (lowest)	12,194 (21.3)	13,334 (21.9)	12,960 (21.8)	13,670 (22.2)
Q2	11,729 (20.4)	12,373 (20.3)	11,908 (20.0)	12,422 (20.1)
Q3	12,660 (22.1)	13,270 (21.8)	13,162 (22.1)	13,288 (21.5)
Q4	11,841 (20.6)	12,421 (20.4)	12,135 (20.4)	12,622 (20.5)
Q5 (highest)	8,957 (15.6)	9,611 (15.8)	9,330 (15.7)	9,665 (15.7)
**Ontario Transitional Health Regions**				
Central	37,695 (65.7)	39,897 (65.4)	38,967 (65.5)	40,513 (65.7)
East	7,887 (13.7)	8,535 (14.0)	8,343 (14.0)	8,673 (14.1)
North	*8-12 (0.0)	14 (0.0)	11 (0.0)	8 (0.0)
Toronto	11,615 (20.2)	12,366 (20.3)	11,991 (20.2)	12,268 (19.9)
West	171 (0.3)	186 (0.3)	165 (0.3)	199 (0.3)
Missing Data	*1-5 (0.0)	11 (0.0)	13 (0.0)	6 (0.0)
**Primary Care Provider (yes)**	56,166 (97.9)	60,378 (99.0)	58,850 (98.9)	60,958 (98.9)
**Child Health Provider**				
Family Physician / General Practitioner	30,839 (53.7)	34,345 (56.3)	33,648 (56.6)	34,675 (56.2)
Pediatrician	25,327 (44.1)	26,033 (42.7)	25,202 (42.4)	26,283 (42.6)

No Primary Care Provider	1,215 (2.1)	631 (1.0)	640 (1.1)	709 (1.1)
**Continuity of Care**				
Yes	37,878 (66.0)	33,762 (55.3)	33,746 (56.7)	34,503 (56.0)

SD = standard deviation; IQR = inter quartile range

* Suppression of data due to cell sizes less than six, to ensure no risk of re-identification of patients per institutional policy.

[1]Continuity of care was defined by having ≥76% of visits to the assigned primary care provider

Table 7. Comparison of immunizations and well-child visits for infants younger than 12 months old who were exposed or unexposed to the pandemic in the Greater Toronto Area (GTA). All data shown as number (%) unless otherwise indicated.

	**Ontario**	
	Exposed N=57,381	Unexposed N=61,009
**Vaccination Outcomes**		
Primary Outcome		
**Received all vaccines[1]**	38,935 (67.9)	40,663 (66.7)
Secondary Outcome		
Number of DTaP-IPV-HiB received [Mean (SD)|Median (IQR)]	2.5 (1.0) | 3 (2-3)	2.4 (1.0) | 3 (2-3)
0	5,684 (9.9)	6,877 (11.3)
1	3,428 (6.0)	3,254 (5.3)
2	7,626 (13.3)	8,184 (13.4)
3 or more	40,643 (70.8)	42,694 (70.0)
Number of Pneu-C-13 received [Mean (SD)|Median (IQR)]	1.7 (0.8) | 2 (2-2)	1.7 (0.8) | 2 (1-2)
0	7,007 (12.2)	8,551 (14.0)
1	6,972 (12.2)	6,910 (11.3)
2 or more	43,402 (75.6)	45,548 (74.7)
**Well-child Visit Outcomes**		
Primary Outcome		
**Received 4 visits[2]**	18,391 (32.1)	30,443 (49.9)
Secondary Outcome		
Number of visits received [Mean (SD)|Median (IQR)]	2.6 (1.8) | 3 (1-4)	3.3 (2.0) | 3 (2-5)
0	10,265 (17.9)	7,979 (13.1)
1	6,792 (11.8)	5,139 (8.4)
2 or more	40,324 (70.3)	47,891 (78.5)
3 or more	31,799 (55.4)	41,389 (67.8)
4 or more	18,391 (32.1)	30,443 (49.9)

[1]Infants were considered to have received all vaccinations if they received three doses of DTaP-IPV-HiB two doses of Pneu-C-13, in Ontario prior to 12 months of age.
[2]Received all recommended well-child visits prior to 12 months of age.

Table 8. Comparison of immunizations and well-child visits of children between the ages of 12 to 24 months who were exposed or unexposed to the pandemic in the Greater Toronto Area (GTA), Ontario, Canada. All data shown as number (%) unless otherwise indicated.

	**Ontario**			
	Exposed N=59,490		Unexposed N=61,667	
**Vaccination Outcomes**				
Primary Outcome				
**Received all vaccinations[1]**	31,387 (52.8)		33,616 (54.5)	
Secondary Outcome				
**Number of Doses Received Mean (SD)**				
DTaP-IPV-HiB	0.74 (0.50)		0.76 (0.49)	
Pneu-C-13	0.78 (0.45)		0.78 (0.46)	
Men-C-C	0.78 (0.45)		0.79 (0.45)	
MMR	0.79 (0.43)		0.81 (0.43)	
Varicella	0.72 (0.47)		0.74 (0.46)	
**Well-child Visit Outcomes**				
Primary Outcome				
**Received recommended visits[2]**				
3 or more	17,306 (29.1)		24,202 (39.2)	
2 or more	32,371 (54.4)		39,716 (64.4)	
Enhanced 18 month	32,581 (54.8)		39,832 (64.6)	
Secondary Outcome				
**Number of visits received [Mean (SD)|Median (IQR)]**				
Total	1.7 (1.2)	2 (1-3)	1.9 (1.2)	2 (1-3)
0	12,499 (21.0)		9,221 (15.0)	
1	14,620 (24.6)		12,730 (20.6)	
2 or more	15,065 (25.3)		15,514 (25.2)	
3 or more	17,306 (29.1)		24,202 (39.2)	

[1]A child was considered to have received all vaccinations if they received one dose of each Pneu-C-13, Men-C-C, MMR, Varicella between the age of 12 and 24 months for Ontario.

[2]Received well-child visits between the ages of 12 and 24 months. In Ontario only, an 'enhanced 18-month visit' is recommended. The 18-month visit is included in the total recommended visits.

Table 9. Receipt of vaccinations and well-child visits in Ontario, Canada, comparing infants less than 12 months of age post-pandemic and children between 12 to 24 months of age post-pandemic to their counterparts during the pre-pandemic period. The Poisson model was adjusted for maternal age at delivery, parity, neighbourhood income, rurality, health region, and health care provider. In Ontario, received all vaccinations refers to having received one dose of each DTaP, Pneu-C-13, Men-C-C, MMR, and Varicella between the ages of 12 to 24 months.

	**Ontario**			
	**Infants (<12 months old)**		**Children (12 – 24 months old)**	
	Unadjusted Relative Risk (95% CI)	Adjusted Relative Risk (95% CI)	Unadjusted Relative Risk (95% CI)	Adjusted Relative Risk (95% CI)
**Vaccination Outcomes**				
Primary Outcome				
Received all vaccinations	1.02 (1.01-1.03)	1.02 (1.01-1.03)	0.97 (0.96-0.98)	0.97 (0.95-0.98)
Secondary Outcomes				
Recommended DTaP Vaccination Doses				
1+			0.97 (0.96-0.98)	0.97 (0.96-0.98)
3+	1.01 (1.00-1.02)	1.01 (1.00-1.02)		
Recommended Pneu Vaccination Doses				
1+			1.00 (0.99-1.01)	1.00 (0.99-1.01)
2+	1.01 (1.00-1.02)	1.01 (1.00-1.02)		
Recommended MMR Vaccination				
1+			0.99 (0.98-0.99)	0.98 (0.98-0.99)
Recommended Men C-C Vaccination				
1+			0.99 (0.98-1.00)	0.99 (0.98-0.99)
Recommended Varicella Vaccination				
1+			0.97 (0.96-0.98)	0.97 (0.96-0.98)
**Well-child Visit Outcomes**				
Primary Outcome				
**Received Recommended Visits**				
3 or more			0.74 (0.73-0.75)	0.74 (0.73-0.75)
4 or more	0.64 (0.63-0.65)	0.64 (0.63-0.65)		
Enhanced 18-month visit			0.85 (0.84-0.86)	0.85 (0.84-0.86)
Secondary Outcome				
**Number of Visits Received**				
2 or more	0.90 (0.89-0.90)	0.89 (0.89-0.90)	0.88 (0.87-0.88)	0.87 (0.87-0.88)
3 or more	0.82 (0.81-0.83)	0.81 (0.81-0.82)		
4 or more	0.66 (0.65-0.67)	0.65 (0.64-0.67)		

Abbreviations: DTaP = diphtheria, tetanus, pertussis, polio, and haemophilus influenzae type B, Pneu-C = pneumococcal conjugate 13, Men-C-C =meningococcal type C MMR=measles, mumps and rubella, MMR-V= measles, mumps, and rubella and varicella
