# Supplementary material for: Gaps in childhood immunizations and preventive care visits during the COVID-19 pandemic: a population-based cohort study of children in Ontario and Manitoba, Canada, 2016–2021
Source: Can J Public Health. 2023 Jul 13;114(5):774–86. doi: 10.17269/s41997-023-00797-y (PMC10484833; doi:10.17269/s41997-023-00797-y)
Supplement: Supplementary file 2 — Supplementary file2 (PDF 254 KB) [file 41997_2023_797_MOESM2_ESM.pdf]

Gaps in childhood immunizations and preventative care visits during the COVID-19 pandemic – A population-based cohort study of children in Ontario and Manitoba, Canada, 2016 - 2021

Canadian Journal of Public Health

Andrea Evans[1-3], Alyson L Mahar[4,5], Bhumika Deb[3], Alexa Boblitz[3], Marni Brownell[4-6], Astrid Guttmann[3,8,-13], Therese Stukel[3], Eyal Cohen[3, 8-13], Joykrishna Sarkar[5], Nkiruka Eze[5], Alan Katz[4-6], Tharani Raveendran[9], Natasha Saunders[3,8-13]

[1]Children's Hospital of Eastern Ontario, Ottawa, Canada;
[2]Department of Pediatrics, University of Ottawa, Ottawa, Canada;
[3]ICES, Toronto, Canada
[4]Department of Community Health Sciences, University of Manitoba, Winnipeg, Canada
[5]Manitoba Centre for Health Policy, Winnipeg, Manitoba
[6]Children's Hospital Research Institute of Manitoba, Winnipeg, Manitoba
[7]Department of Family Medicine University of Manitoba, Winnipeg, Manitoba
[8]The Hospital for Sick Children, Toronto, Canada
[9]Child Health Evaluative Sciences, SickKids Research Institute, Toronto, Canada
[10]Department of Pediatrics, University of Toronto, Toronto, Canada
[11]Institute of Health Policy, Management and Evaluation, The University of Toronto, Toronto, Canada
[12]Temerty Faculty of Medicine, University of Toronto, Toronto, Canada
[13]Edwin S.H. Leong Centre for Healthy Children, University of Toronto, Toronto, Canada

Corresponding author: Natasha Saunders, Natasha.saunders@sickkids.ca,
T: 416-813-7654 x 203076

Fig 1

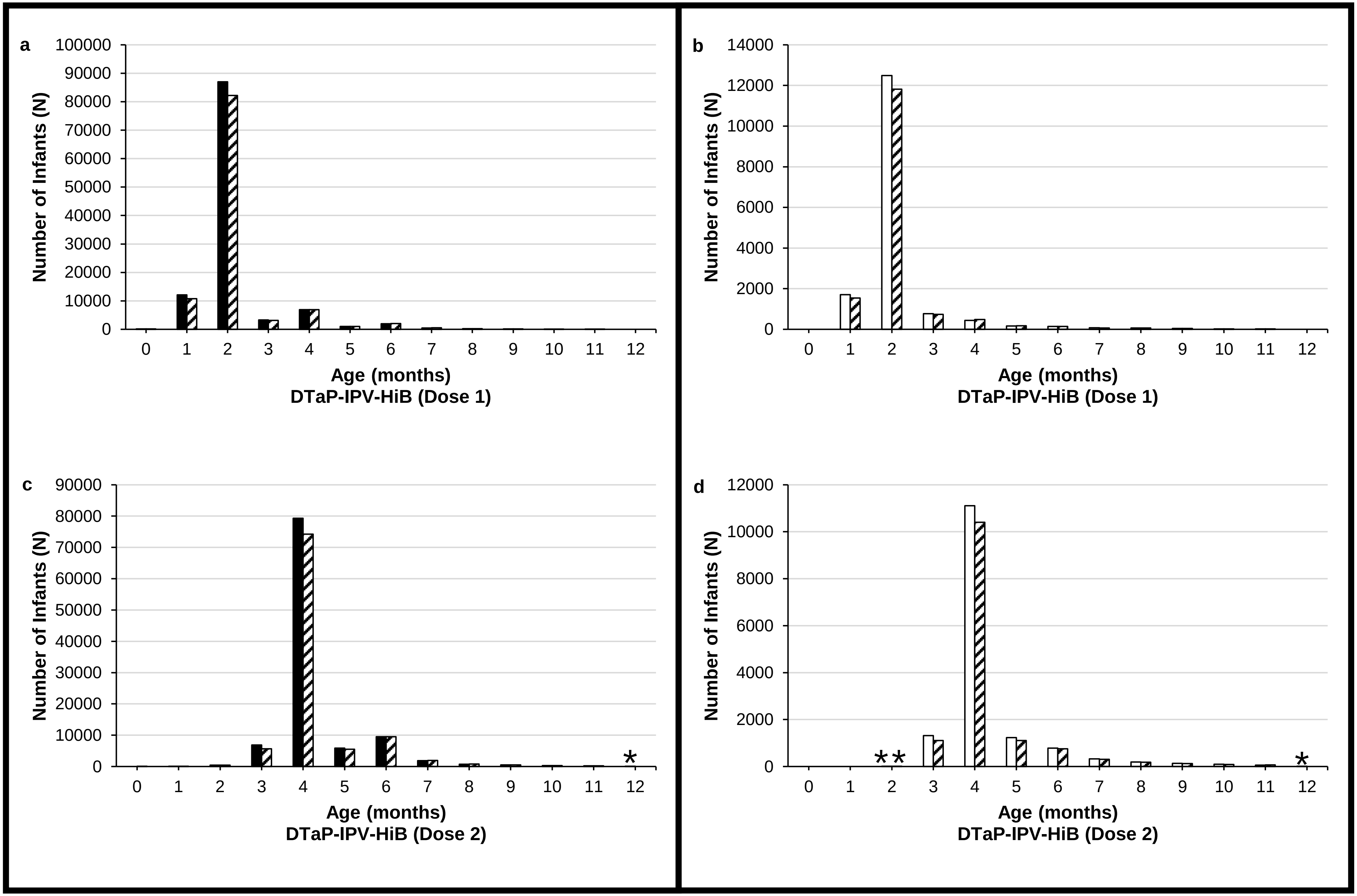
a
Number of Infants (N)
Age (months)
DTaP-IPV-HiB (Dose 1)
b
Number of Infants (N)
Age (months)
DTaP-IPV-HiB (Dose 1)
c
Number of Infants (N)
Age (months)
DTaP-IPV-HiB (Dose 2)
*
d
Number of Infants (N)
Age (months)
DTaP-IPV-HiB (Dose 2)
**
*

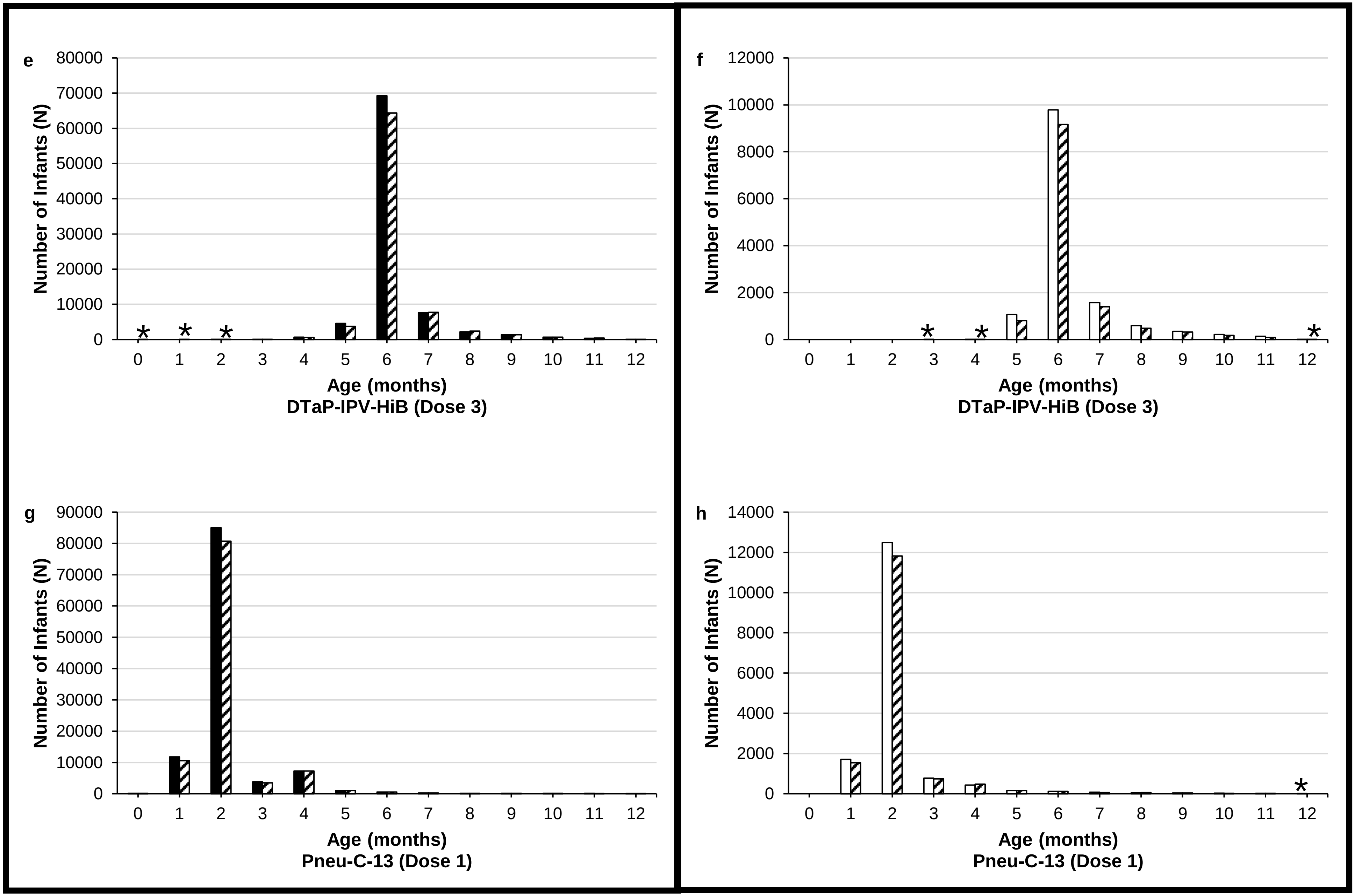

e
Number of Infants (N)
80000
70000
60000
50000
40000
30000
20000
10000
0
*
*
*
0 1 2 3 4 5 6 7 8 9 10 11 12
Age (months)
DTaP-IPV-HiB (Dose 3)
f
Number of Infants (N)
12000
10000
8000
6000
4000
2000
0
*
*
*
0 1 2 3 4 5 6 7 8 9 10 11 12
Age (months)
DTaP-IPV-HiB (Dose 3)
g
Number of Infants (N)
90000
80000
70000
60000
50000
40000
30000
20000
10000
0
0 1 2 3 4 5 6 7 8 9 10 11 12
Age (months)
Pneu-C-13 (Dose 1)
h
Number of Infants (N)
14000
12000
10000
8000
6000
4000
2000
0
*
0 1 2 3 4 5 6 7 8 9 10 11 12
Age (months)
Pneu-C-13 (Dose 1)

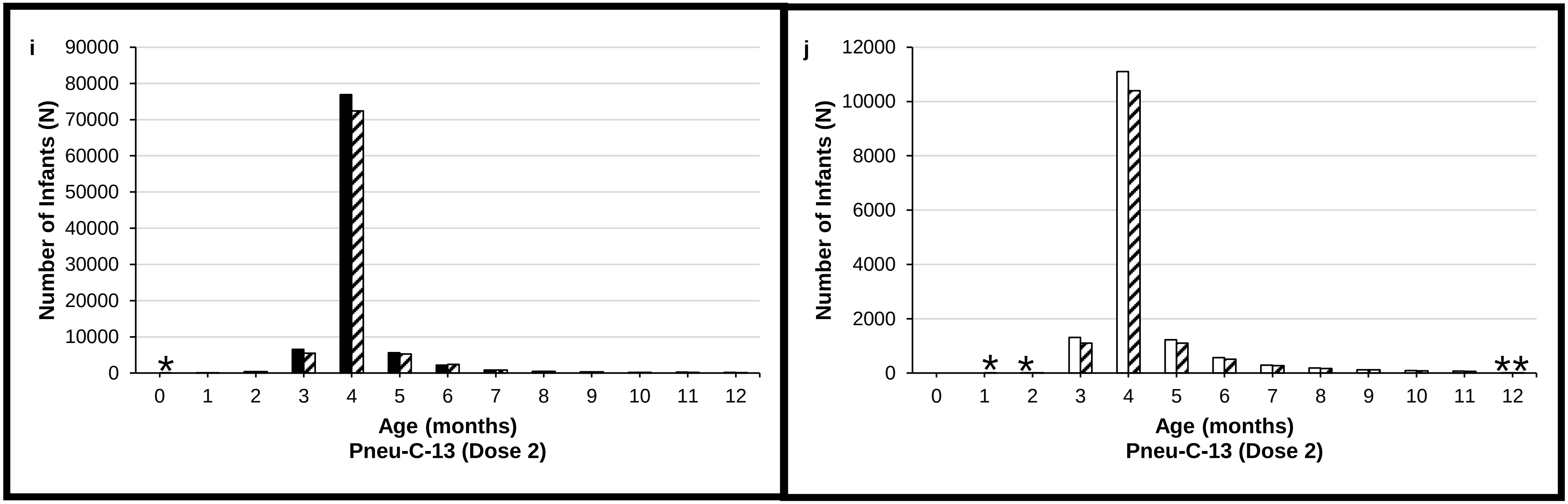

Unexposed cohort is illustrated in black (Ontario) and white (Manitoba), exposed cohort is illustrated with lined pattern.

*Suppression of data due to cell sizes less than six to ensure no risk of re-identification of patients per institutional policy.

Fig 2

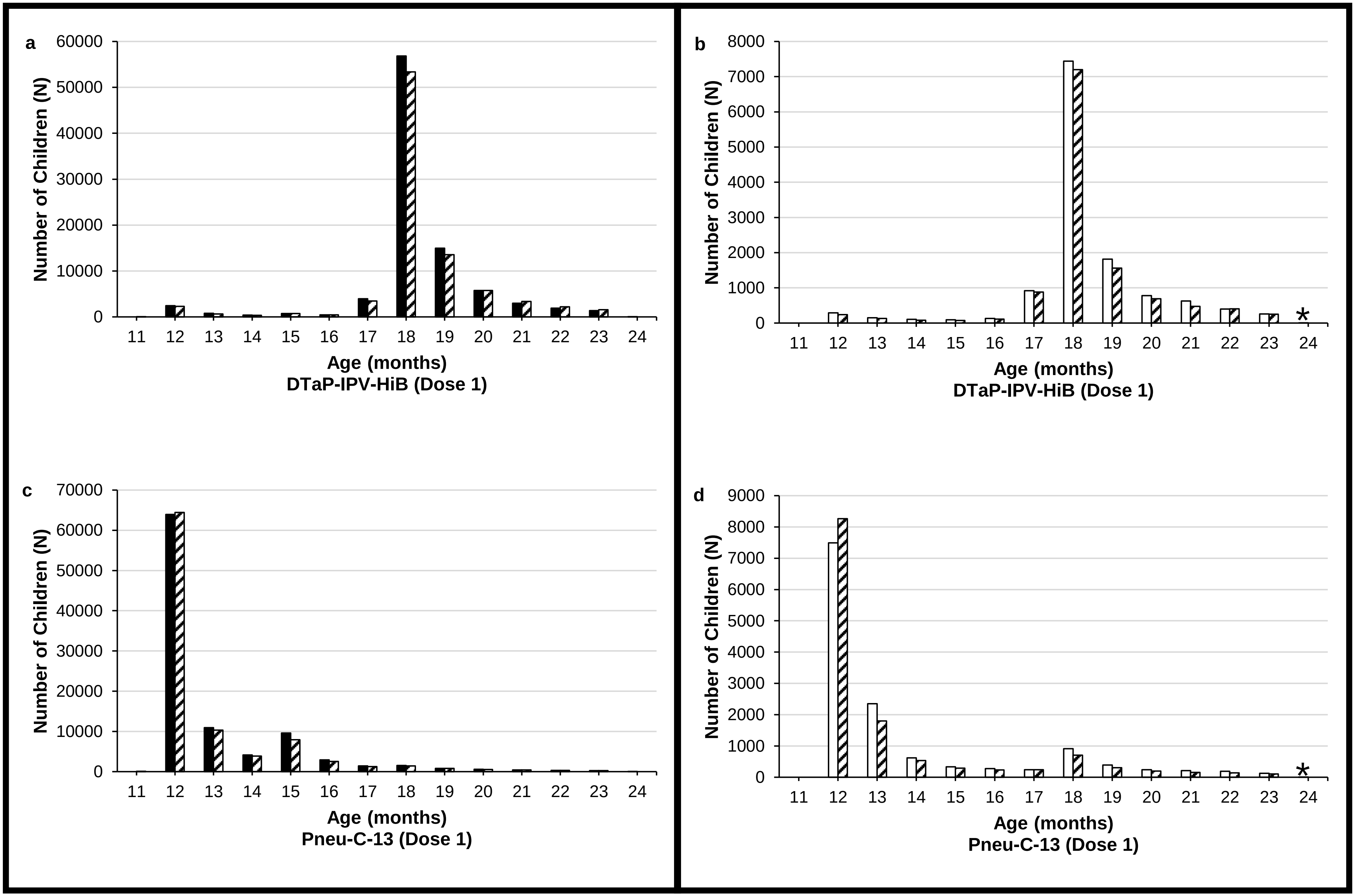
a
b
c
d
Number of Children (N)
60000
50000
40000
30000
20000
10000
0
8000
7000
6000
5000
4000
3000
2000
1000
70000
9000
11 12 13 14 15 16 17 18 19 20 21 22 23 24
Age (months)
DTaP-IPV-HiB (Dose 1)
Pneu-C-13 (Dose 1)
*

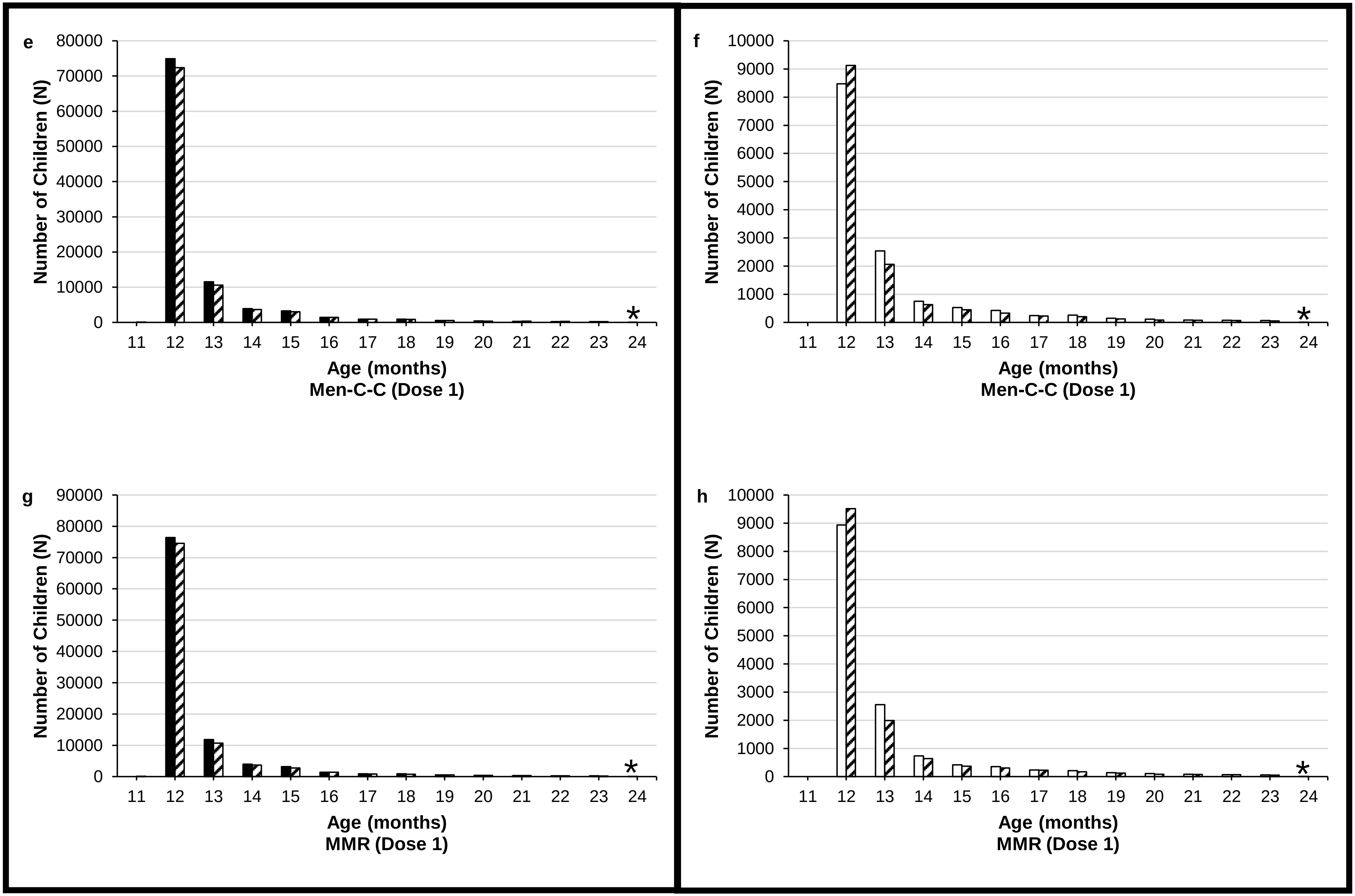

Unexposed cohort is illustrated in black (Ontario) and white (Manitoba), exposed cohort is illustrated with lined pattern.

*Suppression of data due to cell sizes less than six, to ensure no risk of re-identification of patients per institutional policy.
